# Predictive performance of lifestyle-informed multimodal brain features for cognition in healthy older adults

**DOI:** 10.1038/s41514-026-00558-4

**Published:** 2026-11-14

**Authors:** Mingxian Zhang, Nora Bittner, Camilla Mendl-Heinisch, Tatiana Miller, Susanne Moebus, Nico Dragano, Svenja Caspers

**Affiliations:** 1 Institute of Neuroscience and Medicine (INM-1), Research Centre Jülich, Jülich, Germany; 2 Institute for Anatomy I, Medical Faculty & University Hospital Düsseldorf, Heinrich Heine University Düsseldorf, Düsseldorf, Germany; 3 Institute for Urban Public Health, University of Duisburg-Essen, Essen, Germany; 4 Institute of Medical Sociology, Medical Faculty & University Hospital Düsseldorf, Heinrich Heine University Düsseldorf, Düsseldorf, Germany

**Keywords:** Neuroscience, Psychology, Psychology

## Abstract

Identifying neuroimaging markers of cognitive performance in healthy aging is challenging due to heterogeneity in brain and cognitive characteristics. Among the factors relevant to that heterogeneity are lifestyle choices, of which each individual factor explains only a very small amount of variance in brain and cognition. This challenges current approaches for the prediction of cognitive outcomes. We examined whether brain features associated with an overall lifestyle profile (BF-Life_**multi**_) provide predictive information for 13 cognitive outcomes compared with those related to single lifestyle factors (BF-Life_**single**_), in 774 participants from the 1000BRAINS study (age: 67.0 ± 6.7 years), cross-sectionally and longitudinally (4-year follow-up). The BF-Life_**multi**_ model showed modest domain-specific prediction, with the highest performance for baseline figural memory, primarily involving regions within the salience/ventral attention, default mode, somatomotor, and visual networks. These models did not consistently outperform data-driven models based on general brain features with shared variance alone. Including demographic variables improved prediction, whereas individual lifestyle factors added little benefit. No model reliably predicted longitudinal cognitive outcomes. Overall, lifestyle-related brain differences can contribute domain-specific complementary information for cognitive performance prediction in aging, but are not generalizable across domains and for capturing intraindividual trajectories.

## Introduction

Global demographic shifts and increasing life expectancy highlight the need to better understand mechanisms and factors influencing brain aging. This lays the grounds for detecting early cognitive decline and consecutively identifying potentially relevant biomarkers. Biomarkers based on blood, cerebrospinal fluid, or genetic measures have attracted attention in that regard^1^, but are limited in capturing specific, regionally distributed effects on brain structure and network architecture associated with specific cognitive outcomes. Therefore, neuroimaging-derived biomarkers came into focus, which allow identifying subtle age-related differences in brain architecture in healthy adults, as such potentially enabling a biologically mechanistic understanding of cognitive aging.

Age-related differences in MRI regarding brain structure, white-matter integrity, and functional network organization are associated with cognitive performance across domains such as episodic memory and executive function^2–4^. Each aspect of brain organization at the systems level captures complementary aspects of brain organization; hence, no single modality can fully account for the substantial heterogeneity observed in cognitive aging^5^. Consequently, multimodal approaches are needed for a comprehensive characterization of structural and functional differences underlying cognitive aging^6^. However, combining multiple modalities results in high-dimensional, strongly interdependent, and nonlinear data, which limits the ability of conventional linear or low-dimensional multivariate methods to explain or predict individual cognitive variation. Therefore, in such data, regularized and cross-validated machine learning (ML) methods provide a promising new approach to leverage distributed cross-modal multivariate structure, may capture limited nonlinear patterns, and have, in some large-scale studies, been shown to yield small but reproducible improvements in predictive performance over conventional linear models^7–9^. While most studies have focused on cross-sectional prediction, i.e., cognitive function acquired at the same timepoint as brain characteristics, it remains particularly important to determine whether future intraindividual cognitive performance metrics at a later timepoint can be predicted similarly well^10^.

Yet, even with multimodal neuroimaging, explained variance for the prediction of cognitive targets in healthy individuals, including older adults, rarely exceeds 10–15%. For example, prior studies reported explained variance <10% for the prediction of overall cognition in young adults (HCP, *N* = 1050)^9^, up to 12% for non-verbal function^7^, and up to 14% for global cognition in older adults (1000BRAINS, *N* = 594–634)^11^. Large-scale simulations suggest that increasing sample size to one million can improve prediction, yet explained variance remains modest, peaking around 13% (UK Biobank)^8^. A comparable finding was made by a UK Biobank study (*N* = 20,000), indicating that prediction performance is largely independent of sample size as soon as a certain sample size is reached^12^.

These modest predictive outcomes likely reflect the inherent complexity and inter-individual variability of brain aging, which may be influenced by non-brain factors, such as genetics, health conditions (e.g., obesity), lifestyle (e.g., smoking), and various social factors (e.g., social integration). For example, Smith et al. identified 62 distinct aging trajectories, some of which were modulated by lifestyle factors such as physical activity and diet (UK Biobank, *N* = 21,407, aged 40–69 years)^13^. Lifestyle, here understood as those factors influencing a human’s health (e.g., nutrition, sleeping behavior, physical activity, and smoking, alcohol consumption), has gained particular interest in the search for personalized preventive strategies because it is modifiable and interacts dynamically with brain aging^14^. Importantly, lifestyle factors may not only influence current brain properties and cognitive performance but also relate to future cognitive trajectories, making them key targets for understanding and predicting longitudinal changes in cognition.

The Scaffolding Theory of Aging and Cognition—revised (STAC–r) provides a useful framework by positing that age-related neural decline is accompanied by compensatory neural scaffolding, which is associated with lifestyle factors and cognitive performance across aging^15^. Lifestyle factors such as physical activity and stair climbing may support brain health^16,17^. Psychosocial factors, like social support and integration, could also influence cognitive health, being linked to larger brain volumes, slower volume loss, and enhanced functional connectivity^18,19^. Conversely, factors such as sedentary behavior, smoking, and alcohol consumption have been associated with brain changes that might contribute to cognitive decline—for instance, each packyear of smoking could increase brain age by ~0.6 months^20^, while sedentary behavior was associated with lower cognitive performance^21^, and higher alcohol intake was associated with lower gray matter volume^22^. A lack of physical and social interaction was also linked to weaker connectivity in memory-related brain regions, while smoking mainly affected attention-related regions^23^. These findings suggest that lifestyle factors may partly contribute to individual differences in brain and cognitive aging.

Although prior studies have mainly examined individual lifestyle factors in relation to brain aging^22,24^, lifestyle and psychosocial factors, such as alcohol consumption and social integration, are often interconnected, and their combined effects may differ from the sum of individual contributions. Some studies have created composite lifestyle measures, including risk scores from linearly transformed variables^20,25^ or indices based on the frequency of healthy behaviors^23,26^. However, these measures typically assume equal and independent contributions, potentially overlooking interactions. In contrast, clustering analysis groups participants based on their multidimensional lifestyle profiles—for example, identifying a group of individuals who smoke while also being socially and physically active—thereby capturing the co-occurrence of behaviors that may have opposing effects on brain reserve and offering a more ecologically valid approach to modeling lifestyle patterns in relation to brain health^27^. We therefore assumed that identifying brain features associated with these lifestyle-profile groups may provide an interpretable way to test whether lifestyle-related brain variation carries cognitive-predictive information, complementary to fully data-driven feature-reduction methods such as principal component analysis (PCA), where individual components combine information across multiple brain features and may therefore be less directly interpretable in terms of specific anatomical or functional brain features and their relationships with lifestyle^28^.

Although lifestyle and psychosocial factors (hereinafter collectively referred to as lifestyle factors) have been associated with brain architecture and cognition, it remains unclear whether brain features related to multidimensional lifestyle profiles provide useful information for predicting cognitive performance. We therefore tested the predictive performance of multiple lifestyle-informed multimodal brain features and compared their performance with brain features related to individual lifestyle factors and with brain features selected without lifestyle information using fully data-driven approaches. We additionally examined prediction from lifestyle variables alone and whether adding demographic and lifestyle variables to the brain-based models improved prediction. Finally, we evaluated whether the observed cross-sectional predictive patterns extended to longitudinal cognitive outcomes and global cognitive performance.

## Results

### Demographic and neuropsychological measurements

Basic information of the whole sample (*N* = 774, mean age =  67.0 ± 6.7 years) and its three sub-sample sets are listed in Table 1. Details of the baseline and follow-up cognitive performance of the participants in TEST_LONG_ were listed in the Suppl. Table 3.

**Table 1:** Basic information about participants

	Mean (SD)				Statistic	
	Whole sample (*N* = 774)	DISCOVERY (*n* = 453)	TEST_CROSS_ (*n* = 211)	TEST_LONG_ (*n* = 110)	ᵡ^2^/*t/F* value	*p* value
**Demographics**						
Age^a^	67.0 (6.7)	67.7 (6.6)	66.3 (7.1)	65.3 (6.2)	7.020	**<0.001**
Sex^b^	female, *n* = 357 (46.1%)	female, *n* = 213 (47.0%)	female, *n* = 100 (47.4%)	female, *n* = 44 (40.0%)	1.943	0.378
Education^a^	6.48 (1.96)	6.32 (1.94)	6.71 (1.96)	6.72 (1.96)	3.807	**0.023**
**Lifestyle and psychosocial variables**						
Packyears^c^	/	13.7 (20.64)	/	13.84 (20.43)	−0.832	0.406
Alcohol^c^	/	10.57 (18.01)	/	11.47 (18.47)	−0.649	0.516
Physical activity^c^	/	45.44 (53.77)	/	44.77 (41.24)	−0.961	0.336
Stairs^c^	/	6.04 (8.03)	/	7.28 (11.14)	−1.846	0.065
Social integration^c^	/	12.82 (6.09)	/	13.77 (7.13)	−1.299	0.194
Visit_count^c^	/	6.10 (4.31)	/	6.76 (5.13)	−1.062	0.288
Sporty_count^c^	/	2.53 (1.4)	/	2.48 (1.21)	−0.111	0.911
Physical_count^c^	/	0.12 (0.42)	/	0.11 (0.37)	−0.005	0.996
**Cognitive variables**						
Cognitive status^a^	14.82 (2.43)	14.76 (2.38)	14.89 (2.53)	14.93 (2.47)	0.326	0.722
Language comprehension^a^	30.93 (4.93)	30.79 (5.14)	31.0 (4.93)	31.36 (4.02)	0.633	0.531
Nomination^a^	14.51 (0.84)	14.51 (0.80)	14.46 (0.99)	14.59 (0.69)	0.814	0.444
Figural memory^a^	8.72 (4.23)	8.17 (4.18)	9.26 (4.42)	9.94 (3.70)	10.35	**<0.001**
Verbal WM^a^	4.70 (1.10)	4.64 (1.09)	4.86 (1.19)	4.65 (0.93)	3.088	**0.046**
Phonemic fluency^a^	18.66 (6.01)	19.00 (6.12)	17.98 (5.59)	18.58 (6.26)	2.066	0.127
Phonemic switch^a^	18.90 (5.96)	18.67 (5.88)	19.30 (6.12)	19.05 (5.99)	0.833	0.435
Semantic fluency^a^	23.60 (6.74)	23.25 (6.62)	24.36 (6.78)	23.61 (7.06)	1.978	0.139
Semantic switch^a^	19.86 (4.57)	19.47 (4.69)	20.70 (4.20)	19.86 (4.57)	5.281	**0.005**
Visuospatial WM^a^	4.70 (1.05)	4.61 (1.06)	4.82 (1.01)	4.78 (1.10)	3.258	**0.039**
Episodic memory^a^	60.76 (11.63)	59.86 (11.49)	61.80 (11.66)	62.49 (11.90)	3.431	**0.033**
Long-term memory^a^	10.74 (2.63)	10.67 (2.62)	10.76 (2.55)	10.95 (2.80)	0.498	0.608
Executive control^a^	2.40 (0.88)	2.46 (0.96)	2.30 (0.75)	2.38 (0.74)	2.375	0.094

Values are shown as mean (standard deviation). Significant group differences are indicated in bold.

*Packyears* packyears of smoking, *Alcohol* alcohol consumption, *Physical activity* measured by metabolic equivalent of task, *Stairs* flights of stairs climbed, *Visit_count* count of visits from children, relatives, and friends, *Sporty_count* count of sporty activities, *Physical_count* count of physical activities, *WM* working memory.

^a^ Univariate analysis of variance.

^b^ Pearson chi-square.

^c^ Mann–Whitney.

### Lifestyle subgroups and lifestyle-related brain features

A cluster solution of *k* = 5 was selected for further analysis to allow for a biologically meaningful representation of lifestyle profiles (SW/SB ratio = 0.035, *R*^*2*^ = 0.967, Silhouette score = 0.107, DBI = 3.117; see Suppl. Table 5).

**Fig. 1:**
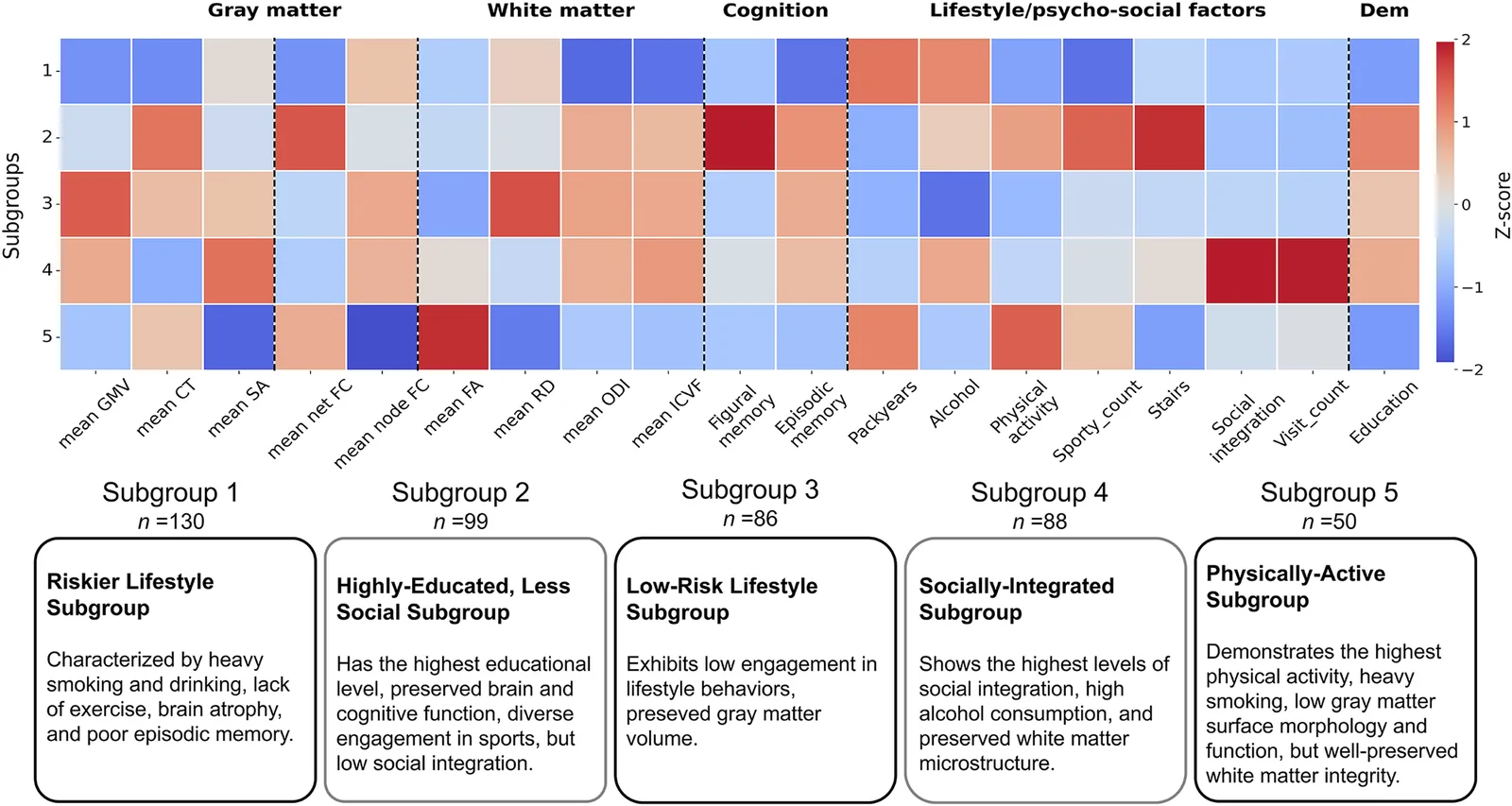
Post hoc characterization of the five lifestyle subgroups identified by clustering. Subgroups were derived from the eight lifestyle/psychosocial variables; demographic, cognitive, and summarized brain measures are shown only to characterize the resulting subgroups after cluster assignment. Top: Variables were averaged within subgroups, normalized, and visualized using colors ranging from red (high) to blue (low). Only variables with significant subgroup differences are shown. For example, subgroup 1 had the lowest mean GMV but the highest packyears of smoking and alcohol consumption. Bottom: Brief summary of the five subgroups. Dem demographics, Stairs flights of stairs, Visit_count visits from children/relatives/friends, Sporty_count count of sporty activities, GMV gray matter volume, CT cortical thickness, SA surface area, FA fractional anisotropy, RD radial diffusivity, ICVF intracellular volume fraction, ODI orientation dispersion index, FC functional connectivity, net FC network-wise FC-related metrics, node FC node-wise functional segregation.

The five lifestyle subgroups showed distinct post hoc profiles across lifestyle, demographic, cognitive, and summarized brain measures, including characteristics beyond the lifestyle indicators used for clustering (Fig. 1, Suppl. Table 6, and Suppl. Figs. 1 and 2). The *Riskier Lifestyle Subgroup* (subgroup 1) exhibited the least healthy overall profile and the poorest episodic memory. In contrast, the *Highly-Educated, Less Social Subgroup* (subgroup 2) demonstrated the highest performance in both domains with relatively low psychosocial factors. Subgroups 3–5 exhibited mixed patterns of lifestyle, cognitive, and brain profiles (see Fig. 1, Suppl. Table 6, and Suppl. Figs. 1 and 2).

In our main analyses, feature selection identified 97 brain features that differed significantly in at least one pairwise comparison of the 5 subgroups. These were primarily GMV, CT, SA, ICVF, and node-wise functional connectivity segregation, distributed mainly across the visual, default mode, salience/ventral attention, dorsal attention, and somatomotor networks. White matter features were mainly in association (e.g., cingulum bundle) and projection fibers (e.g., corona radiata) (Suppl. Fig. 3). The *Riskier Lifestyle Subgroup* (subgroup 1) generally had lower mean values in GMV, CT, ODI, and ICVF and lower network-wise FC strength in the default mode network as compared to the other groups (Suppl. Table 6 and Suppl. Fig. 1B).

All 97 identified brain features were used as input for the BF-Life_**multi**_ model.

### Predictive performance and model comparisons

In the first step of our main analyses, predictive performance of the BF-Life_**multi**_ brain features was evaluated. Overall, BF-Life_**multi**_ showed modest domain-specific prediction for figural memory in DISCOVERY and TEST_CROSS_, but not for TEST_LONG_. Detailed results are given below.

**Fig. 2:**
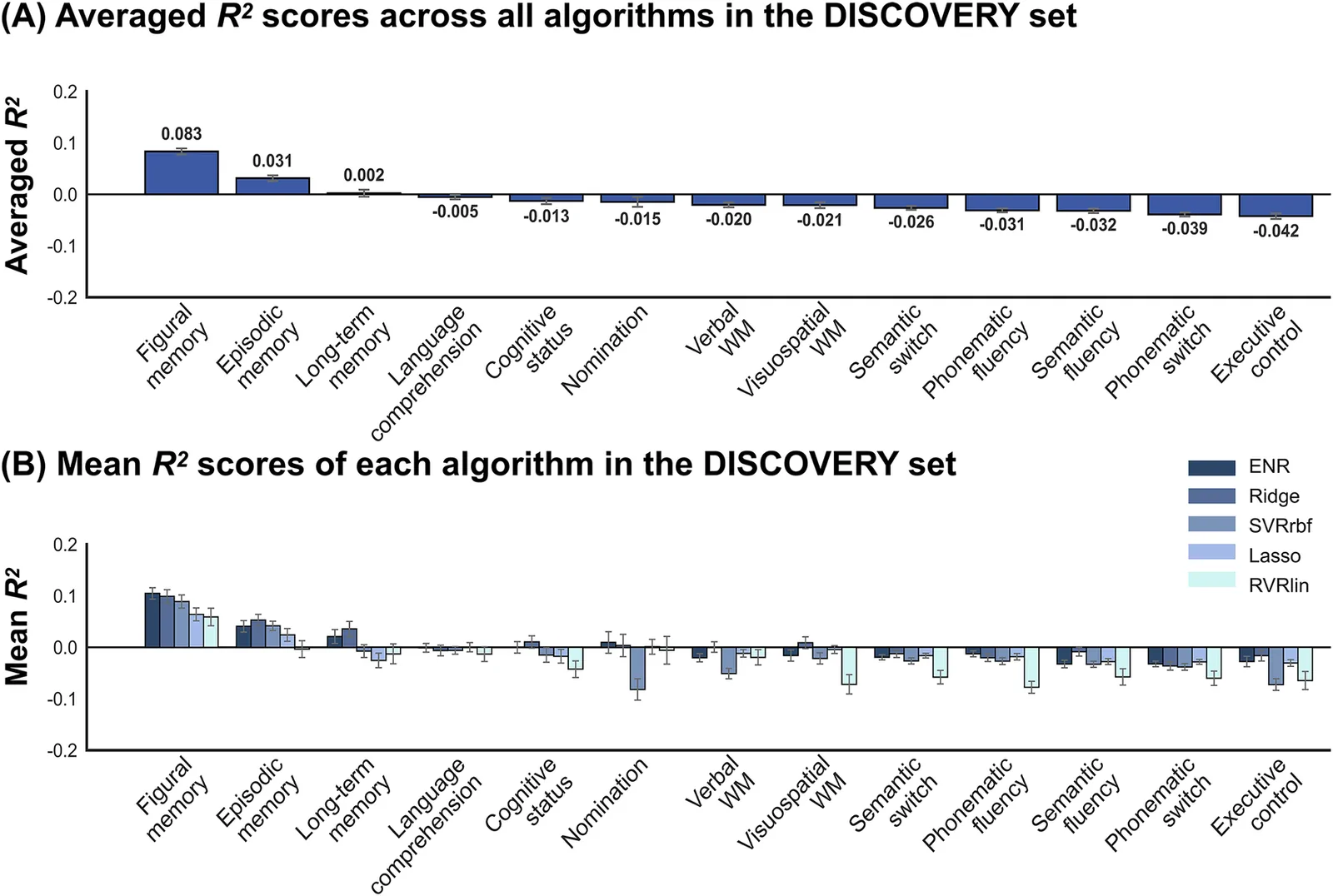
Predictive performance of the BF-Life_multi_ model across cognitive tests in DISCOVERY. **A** Averaged *R²* scores across five algorithms for each cognitive target, shown in descending order. **B** Mean *R²* scores of individual algorithms for the same 13 cognitive targets, displayed in the same order. BF-Life_**multi**_ was built based on a *k* = 5 clustering solution. Abbreviations: BF-Life_**multi**_, brain features derived from group contrasts based on multiple lifestyle profiles; *k* cluster number, WM working memory, ENR elastic net regression, Ridge ridge regression, SVRrbf support vector regression with RBF kernel, Lasso least absolute shrinkage and selection operator regression, RVRlin relevance vector regression with linear kernel.

In DISCOVERY, which was used for model development and internal screening, the BF-Life_**multi**_ models showed positive averaged *R²* values for figural (averaged *R²* = 0.083), episodic (averaged *R²* = 0.031), and long-term memory (averaged *R²* = 0.002), while the remaining ten cognitive test scores could not be reliably predicted (negative averaged *R²*; Fig. 2). Among the five algorithms, elastic net regression (ENR) showed the highest performance for figural memory and was therefore used for focused model comparisons. All individual performances for these three cognitive targets are provided in the Suppl. Table 7.

**Fig. 3:**
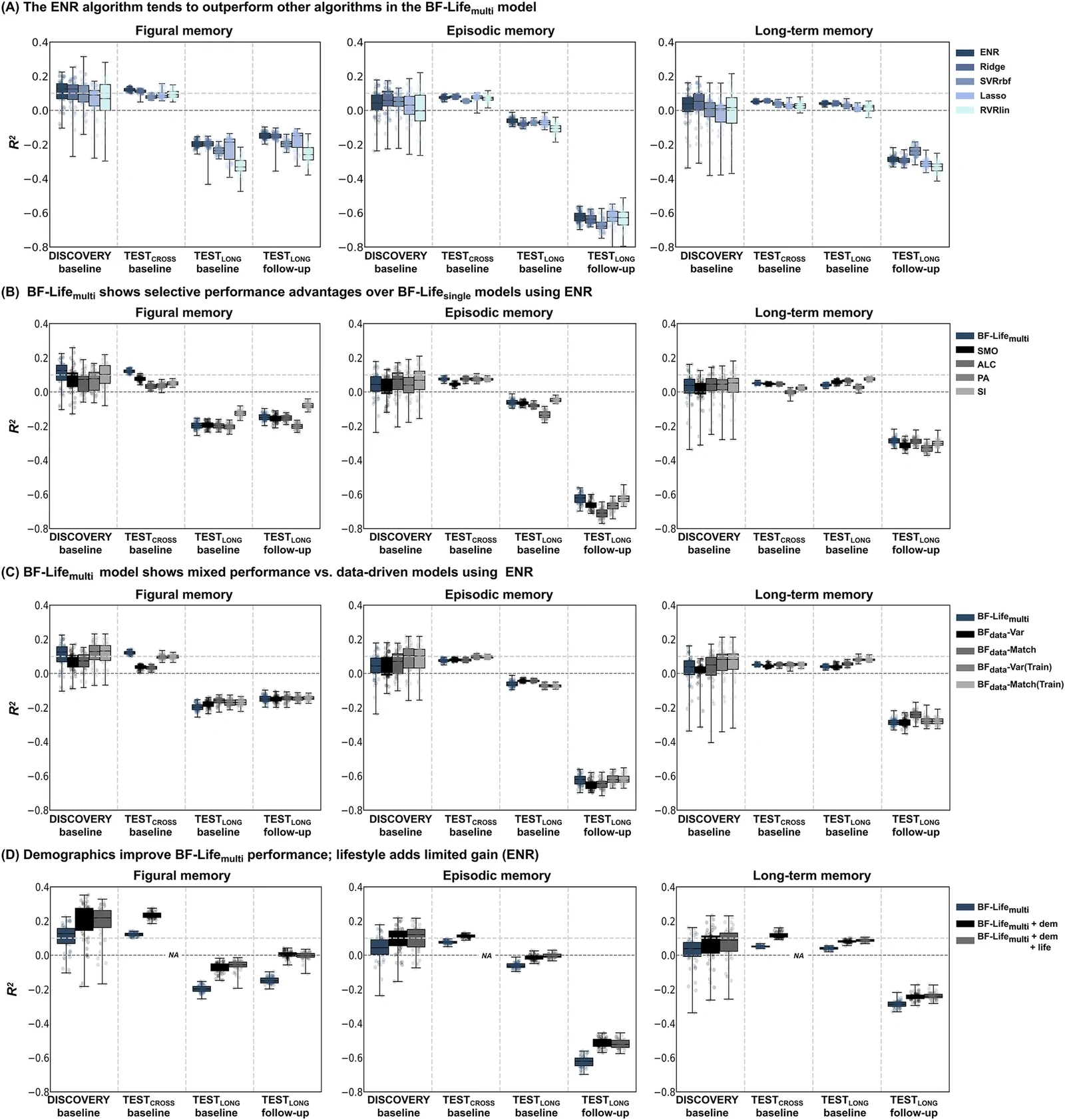
Model performance on predictable targets. **A** Performance of the BF-Life_**multi**_ model predicting figural, episodic, and long-term memory across five algorithms. **B** Comparison with single lifestyle factor models (BF-Life_**single**_) using ENR. **C** Comparison with data-driven models. **D** Comparison with models combining BF-Life_**multi**_ with demographic and lifestyle data. The combined model (BF-Life_**multi**_ +dem+life, light gray) was not available (NA) in TEST_CROSS_ due to missing lifestyle data. BF-Life_**multi**_ was built based on a *k* = 5 clustering solution. The light gray dashed line marks *R²* = 0.10. Abbreviations: BF-Life_**multi**_, brain features derived from group contrasts based on multiple lifestyle profiles; BF-Life_**single**_, brain features derived from group contrasts based on a single lifestyle factor; SMO/ALC/PA/SI: BF-Life_**single**_ models based on smoking, alcohol consumption, physical activity, and social integration, respectively; BF_data_ data-driven PCA brain features, Var 80% variance explained, Match matched feature number, [Train] PCA applied in training set exclusively, +dem, includes demographic data, +dem+life includes demographic and lifestyle data.

Across the three cognitive domains, the BF-Life_**multi**_ model explained a modest proportion of variance in DISCOVERY for figural (median *R*^*2*^ = 0.07–0.13, i.e., 7–13% explained variance), episodic (median *R*^*2*^ = -2.46 × 10^−3^–0.06, i.e., explained variance up to 6%) and long-term memory (median *R*^*2*^ = 0.01–0.05, i.e., 1–5% explained variance, depending on the algorithm; Fig. 3A and Suppl. Table 7). For consistency, model comparisons are reported based on the ENR results.

Using the ENR algorithm, the BF-Life_**multi**_ model showed domain-specific performance patterns (Table 2 and Fig. 3). For prediction of figural memory in DISCOVERY, the BF-Life_**multi**_ model (explained variance of 13%) outperformed the ALC single-factor model and performed comparably to the SMO, PA, and SI models (explained variance up to 10%; Fig. 3B), while showing slightly higher explained variance than BF_data_-Var and BF_data_-Match (explained variance up to 8%) and comparable performance to BF_data_-Var(Train) and BF_data_-Match(Train) (explained variance up to 13%; Fig. 3C). For episodic memory, the BF-Life_**multi**_ model (explained variance of 4%) showed performance similar to that of single lifestyle factor models (Fig. 3B) but was inferior to the data-driven approaches (explained variance up to 10%; Fig. 3C). For long-term memory, BF-Life_**multi**_ (explained variance of 4%) again showed performance comparable to single lifestyle factor models (Fig. 3B) and to BF_data_-Var and BF_data_-Match (Fig. 3C), but remained inferior to BF_data_-Var(Train) and BF_data_-Match(Train) (explained variance up to 8%; Fig. 3C).

**Table 2:** Performance of the 11 models predicting figural, episodic, and long-term memory using the ENR algorithm

Targets	Models	Median *R*^2^				Median MAE			
		DISCOVERY baseline	TEST_CROSS_ baseline	TEST_LONG_ baseline	TEST_LONG_ follow-up	DISCOVERY baseline	TEST_CROSS_ baseline	TEST_LONG_ baseline	TEST_LONG_ follow-up
**Figural memory**	**BF-Life**_**multi**_ **models**								
	*k* = 5^*^	0.13	0.12	−0.20	−0.15	3.16	3.35	3.30	3.32
	**BF-Life**_**single**_ **models**								
	SMO	0.08	0.08	−0.19	−0.15	3.26	3.46	3.28	3.37
	ALC	0.07	0.03	−0.20	−0.15	3.25	3.50	3.33	3.40
	PA	0.08	0.04	−0.20	−0.20	3.25	3.52	3.29	3.46
	SI	0.10	0.05	−0.12	−0.08	3.17	3.49	3.24	3.32
	**Data-driven models**								
	BF_data_-Var	0.08	0.04	−0.18	−0.15	3.22	3.51	3.30	3.39
	BF_data_-Match	0.07	0.04	−0.16	−0.14	3.26	3.51	3.29	3.38
	BF_data_-Var(Train)	0.13	0.10	−0.17	−0.14	3.15	3.37	3.28	3.36
	BF_data_-Match(Train)	0.13	0.10	−0.17	−0.14	3.16	3.36	3.28	3.36
	**Incorporating non-brain data**								
	BF-Life_**multi**_ +dem: *k* = 5	0.21	0.23	−0.07	3.42 × 10^−3^	2.98	3.17	3.09	3.10
	BF-Life_**multi**_ +dem+life: *k* = 5	0.22	NA	−0.06	−5.74 × 10^−4^	3.03	NA	3.09	3.13
**Episodic memory**	**BF-Life**_**multi**_ **models**								
	*k* = 5^*^	0.04	0.07	−0.06	−0.62	8.77	8.93	9.83	10.72
	**BF-Life**_**single**_ **models**								
	SMO	0.04	0.04	−0.07	−0.66	8.89	9.14	9.89	10.98
	ALC	0.07	0.08	−0.08	−0.71	8.82	8.88	9.79	10.92
	PA	0.04	0.08	−0.14	−0.66	8.90	9.02	10.25	11.07
	SI	0.07	0.07	−0.05	−0.62	8.75	9.06	9.77	10.84
	**Data-driven models**								
	BF_data_-Var	0.06	0.08	−0.04	−0.65	8.91	8.95	9.80	10.88
	BF_data_-Match	0.07	0.08	−0.04	−0.64	8.84	8.98	9.80	10.86
	BF_data_-Var(Train)	0.10	0.10	−0.07	−0.62	8.71	8.83	9.84	10.75
	BF_data_-Match(Train)	0.10	0.09	−0.07	−0.62	8.71	8.84	9.83	10.76
	**Incorporating non-brain data**								
	BF-Life_**multi**_ +dem: *k* = 5	0.11	0.11	−0.02	−0.51	8.62	8.76	9.64	10.53
	BF-Life_**multi**_ +dem+life: *k* = 5	0.12	NA	−0.01	−0.52	8.66	NA	9.59	10.55
**Long-term memory**	**BF-Life**_**multi**_ **models**								
	*k* = 5^*^	0.04	0.05	0.04	−0.29	2.00	1.92	2.15	2.21
	**BF-Life**_**single**_ **models**								
	SMO	0.03	0.05	0.06	−0.31	2.02	1.95	2.12	2.21
	ALC	0.05	0.05	0.07	−0.29	2.04	1.95	2.14	2.19
	PA	0.04	−1.15 × 10^−3^	0.03	−0.33	2.02	1.99	2.17	2.23
	SI	0.05	0.02	0.07	−0.30	2.01	1.97	2.12	2.22
	**Data-driven models**								
	BF_data_-Var	0.03	0.04	0.04	−0.29	2.02	1.96	2.16	2.19
	BF_data_-Match	0.05	0.05	0.06	−0.24	2.01	1.93	2.14	2.17
	BF_data_-Var(Train)	0.08	0.05	0.08	−0.28	1.97	1.92	2.12	2.19
	BF_data_-Match(Train)	0.08	0.05	0.08	−0.28	2.00	1.92	2.12	2.19
	**Incorporating non-brain data**								
	BF-Life_multi_ +dem: *k* = 5	0.08	0.11	0.08	−0.24	1.96	1.86	2.09	2.17
	BF-Life_multi_ +dem+life: *k* = 5	0.09	NA	0.09	−0.24	1.93	NA	2.08	2.16

(*) indicates the model used for the main analysis. The combined model (BF-Life_**multi**_ +dem+life) was not available (NA) in TEST_CROSS_ due to missing lifestyle data.

*BF-Life*_*multi*_ brain features derived from group contrasts based on multiple lifestyle profiles, *k* cluster number, *BF-Life*_*single*_ brain features derived from group contrasts based on a single lifestyle factor, *SMO/ALC/PA/SI* BF-Life_single_ models based on smoking, alcohol consumption, physical activity, and social integration, respectively, *PCA* principal component analysis, *BF*_*data*_ data-driven PCA brain features, *Var* 80% variance explained, *Match* matched feature number, *[Train]* PCA applied in training set exclusively, *+dem* includes demographic data, *+dem+life* includes demographic and lifestyle data.

Prediction performance was comparable when applying the BF-Life_**multi**_ model to TEST_CROSS_ for cross-sectional prediction of figural memory using ENR (explained variance of 12%). It showed slightly higher explained variance than both single lifestyle factor and data-driven models (Table 2 and Fig. 3B, C). For episodic memory, the performance of BF-Life_**multi**_ (explained variance of 7%) was higher than that of the SMO single-factor model and comparable to the ALC, PA, and SI single-factor models (Fig. 3B), BF_data_-Var and BF_data_-Match (Fig. 3C), but remained slightly lower than BF_data_-Var(Train) and BF_data_-Match(Train) (explained variance up to 10%; Fig. 3C). For long-term memory, the performance of BF-Life_**multi**_ (explained variance of 5%) was higher than that of the PA and SI single-factor models, while comparable to the SMO and ALC single-factor models (Fig. 3B) as well as the data-driven models (Fig. 3C).

Predictive performance was markedly reduced in TEST_LONG_ across cognitive domains (Table 2 and Fig. 3). For figural and episodic memory, BF-Life_**multi**_ failed to achieve meaningful prediction in both cross-sectional and longitudinal analyses. Similar reductions were observed for the BF-Life_**single**_ and data-driven models (Suppl. Fig. S4). For long-term memory, BF-Life_**multi**_ showed limited cross-sectional performance (explained variance of 4%) but no meaningful longitudinal prediction. In this domain, BF-Life_**multi**_ showed higher explained variance than the PA single-factor model, but lower explained variance than the SMO, ALC, and SI single-factor models (Fig. 3B). Performance of the BF-Life_**multi**_ model was comparable to BF_data_-Var and BF_data_-Match (Fig. 3C), but remained lower than BF_data_-Var(Train) and BF_data_-Match(Train) (explained variance up to 8%; Fig. 3C).

For figural memory, adding demographic variables improved prediction performance in DISCOVERY and TEST_CROSS_, but not in TEST_LONG_ (Table 2 and Fig. 3D). However, adding lifestyle variables on top of this model provided little additional benefit across cognitive domains (Fig. 3D).

### Feature importance for predicting figural memory

Our next main analysis step was feature importance analysis via SHAP focusing on figural memory, specifically for the predictions in TEST_CROSS_ using the ENR algorithm, which showed the most reliable *R²* scores (see Section “Predictive performance and model comparisons”).

From a summary perspective, analysis of TEST_CROSS_ showed distinct feature importance patterns across BF-Life_**multi**_ and BF-Life_**single**_ models. The BF-Life_**multi**_ model showed the largest contributions from structural features (CT, GMV) in SAN, SMN, and DMN, while also incorporating functional and diffusion-derived information (see Suppl. Fig. 5). In contrast, BF-Life_**single**_ models showed more heterogeneous and less integrative patterns: SMO and ALC single-factor models were dominated by structural features with minimal diffusion or functional contributions. Compared with BF-Life_**multi**_, the PA single-factor model showed a similar multimodal profile with less consistent structural dominance and greater diffusion contribution, particularly FA in commissural tracts. The SI single-factor model relied primarily on structural and node-level functional features and diffusion metrics other than FA, with minimal contribution from FA-related metrics.

**Fig. 4:**
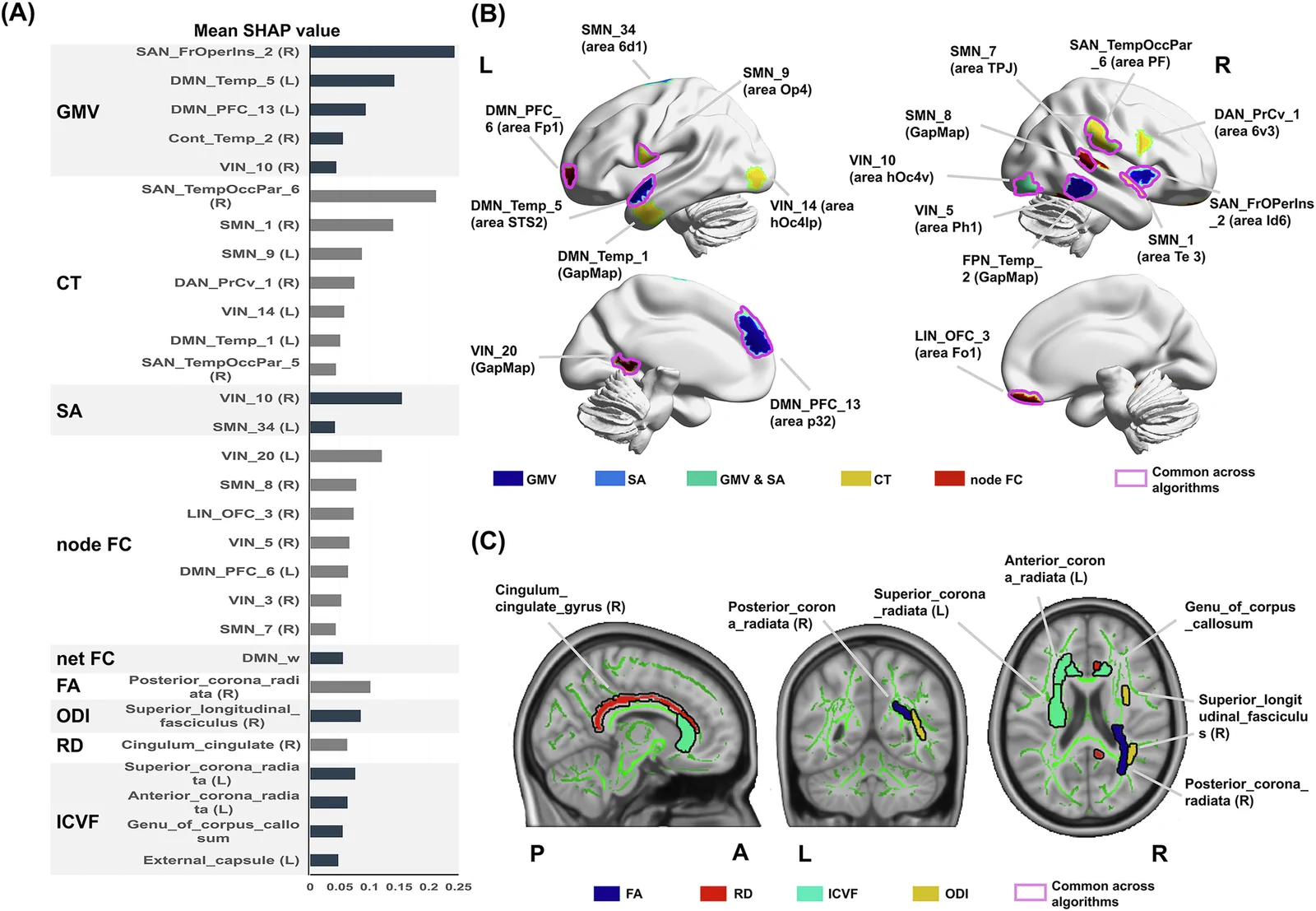
Feature importance in predicting figural memory using the BF-Life_multi_ model with ENR in TEST_CROSS_. **A** Mean SHAP values for the top 30% of features by importance. **B**,** C** Projection of the most important features from gray- and white-matter. BF-Life_**multi**_ was built based on a *k* = 5 clustering solution. Brain regions marked with solid purple lines are consistently important across all five algorithms. Abbreviations: BF-Life_**multi**_, brain features derived from group contrasts based on multiple lifestyle profiles; GMV gray matter volume, CT cortical thickness, SA surface area, FC functional connectivity, net FC network-wise FC-related metrics, node FC node-wise functional segregation, FA fractional anisotropy, RD radial diffusivity, ICVF intracellular volume fraction, ODI orientation dispersion index, L/R left / right hemisphere.

To further characterize these patterns at the feature level, we examined the top 30% of features in the BF-Life_**multi**_ model (Fig. 4; SHAP values 0.042–0.243), which together accounted for 81.27% of the total SHAP contribution. Features that also appeared in the top 30% of any of the other four machine learning algorithms are highlighted (see also Suppl. Table 8).

**Fig. 5:**
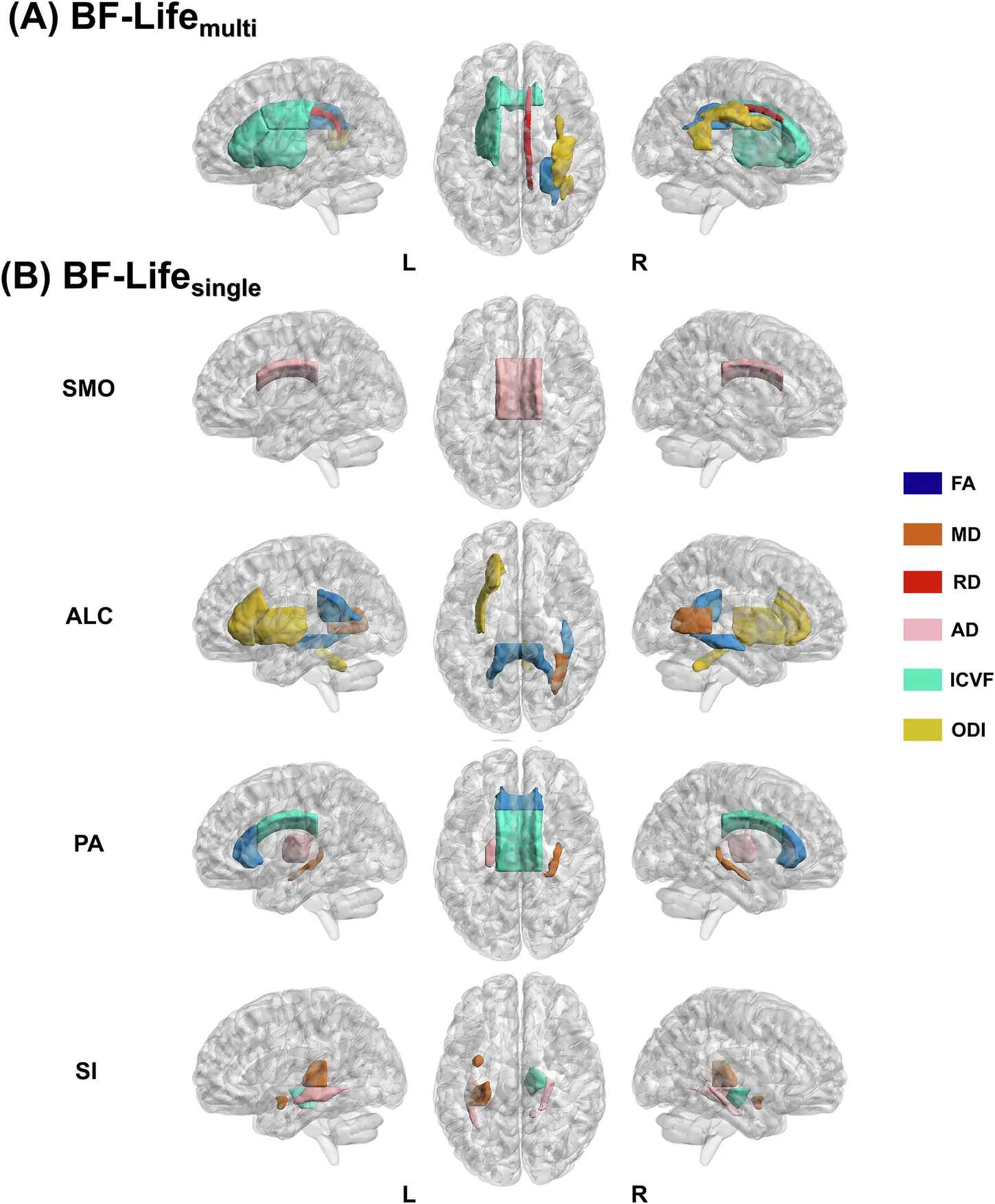
Visualization of white-matter fiber tracts corresponding to the top 30 most important features identified by five ENR models predicting figural memory in TEST_CROSS_. Panel (**A**) shows results from the BF-Life_**multi**_ model, and panel (**B**) shows results from the four BF-Life_**single**_ models. BF-Life_**multi**_ was built based on a *k* = 5 clustering solution. *k* cluster number, BF-Life_**multi**_ brain features derived from group contrasts based on multiple lifestyle profiles, BF-Life_**single**_ brain features derived from group contrasts based on a single lifestyle factor; SMO / ALC / PA / SI: BF-Life_**single**_ models based on smoking, alcohol consumption, physical activity, and social integration, respectively; FA fractional anisotropy, MD mean diffusivity, RD radial diffusivity, AD axial diffusivity, ICVF intracellular volume fraction, ODI orientation dispersion index, L/R left/right hemisphere.

The highest-ranking features were primarily located in the SAN, VIN, DMN, and SMN. The top three SHAP contributors were: (i) GMV in a cluster spanning the right insula and frontal operculum (SAN_FrOperIns_2; area Id6); (ii) CT in the right temporo-parietal junction (SAN_TempOccPar_6; area PF); (iii) SA in the right visual network (VIN_10; area hOc4v). Other highly ranked features encompassed multiple parameters. GMV and CT contributions were observed across several cortical regions, including the left superior temporal sulcus (DMN_Temp_5; area STS2), left medial prefrontal cortex (DMN_PFC_13; area p32), right superior temporal gyrus (SMN_1; area Te3), and left frontal operculum (SMN_9; area Op4). Contributions of node-wise FC segregation were mainly derived from the left visual network (VIN_20), the right superior temporal gyrus (SMN_8), and the right orbitofrontal cortex of the limbic network (LIN_OFC_3; area Fo1). Diffusion-derived white-matter metrics were also among the top features, spanning major projection, association, and commissural fiber tracts such as the posterior and superior corona radiata, superior longitudinal fasciculus, cingulum bundle, anterior corona radiata, genu of the corpus callosum, and external capsule (Fig. 5).

Overlap between BF-Life_**multi**_ and BF-Life_**single**_ models was limited and varied across models, with the greatest overlap observed for the SMO single-factor model, followed by the PA and SI single-factor models, and no shared features for the ALC single-factor model (Suppl. Fig. 6). For diffusion-derived metrics, no identical features were shared, although similar white-matter tracts (e.g., the left anterior corona radiata and left external capsule) were identified using different indices across models (Fig. 5).

### Sensitivity analyses

Across alternative cluster solutions (*k* = 2–4), predictive performance was generally comparable to the main *k* = 5 solution, with *k* = 5 showing slightly higher performance in some cognitive domains. Predictive performance in TEST_LONG_ remained limited across all clustering solutions (Suppl. Table 9 and Suppl. Fig. 4).

Lifestyle-only reference models based on the original eight lifestyle variables showed weak predictive performance for figural memory in DISCOVERY and did not generalize to TEST_LONG_ (Suppl. Table 10 and Suppl. Fig. 7).

For a global cognitive component, performance of the BF-Life_**multi**_ models was similar to that of the BF-Life_**single**_ models, whereas data-driven models showed slightly better performance in DISCOVERY and TEST_CROSS_. Again, predictive performance in TEST_LONG_ remained limited across models. Inclusion of demographic and lifestyle variables further improved the predictive performance of the BF-Life_**multi**_ model (*k* = 5) across datasets (Suppl. Table 11 and Suppl. Fig. 8).

## Discussion

We examined whether brain features derived from differences between groups of participants defined by overall lifestyle profiles provide predictive information for domain-specific cognitive functions. Overall, the model using brain features derived from overall lifestyle profiles showed modest domain-specific predictive performance, with the highest effects observed for figural memory. However, it did not clearly outperform fully data-driven models based on principal component representations of all brain features. Incorporating demographic variables into the BF-Life_**multi**_ model improved prediction performance, whereas further adding lifestyle information itself on top provided little additional benefit. Together, these findings suggest that lifestyle-profile-informed brain features capture a specific portion of cognition-relevant brain variance, although this did not translate into broadly superior predictive performance.

Building on these results, we consider the domain-specific predictive pattern of the BF-Life_**multi**_ model in relation to prior work. Prior studies in comparable aging cohorts using unimodal and multimodal neuroimaging approaches have reported explained variance of up to 12–14% for global cognitive performance^7,11^. In this context, the BF-Life_**multi**_ model showed its strongest predictive performance for figural memory and a global cognitive component, while demonstrating limited predictive sensitivity for other cognitive domains, including working memory, language-related functions, and executive control.

The BF-Life_**multi**_ model did not achieve the higher levels of explained variance (e.g., >20%) reported in studies of young adults using task-based or resting-state FC to predict fluid and crystallized cognitive performance (i.e., intelligence-related measures)^29–31^. However, prediction of cognition in population-based samples across the adult lifespan has generally remained modest^32^. For example, multimodal neuroimaging predicted visual working memory across the adult lifespan in Cam-CAN (~16.2% explained variance; *N* = 547, aged 18–88 years)^33^, while processing speed showed limited predictability using resting-state FC (~8–11%; *N* = 99, aged 62–71 years; Cam-CAN, *N* = 295, aged 25–80 years)^34^. Consistently, a large-scale UK Biobank study explained only a small proportion of variance, with up to ~6% for fluid intelligence and ~2% for neuroticism (*N* = 18,550, aged 44–80 years)^35^.

Although higher predictive performance has been reported in smaller or clinical samples, such as individuals with MCI or Alzheimer’s disease^36–38^, these findings are only partly comparable to population-based studies, given the more specific and pronounced neural changes in clinical cohorts and the increased risk of overfitting in smaller samples^39,40^. In this context, the modest predictive performance observed here is broadly consistent with large-scale population studies such as the UK Biobank^35^ and likely reflects realistic effect sizes in normal aging. Taken together, while the predictive performance of the BF-Life_**multi**_ model in older adults may appear modest compared to studies in young adults or clinical cohorts, its performance, particularly for figural memory and the global cognitive component, remains informative and broadly within the expected range in the context of age-related variability.

Within the present framework, the BF-Life_**multi**_ model showed an advantage over BF-Life_**single**_ models mainly in predicting figural memory. Specifically, in DISCOVERY, the BF-Life_**multi**_ model outperformed the ALC single-factor model. In TEST_CROSS_, the BF-Life_**multi**_ model showed a modest advantage over BF-Life_**single**_ models in predicting figural memory (Fig. 3B). Although the absolute effect sizes were small, integrating multiple lifestyle-related brain features captured lifestyle-informed brain variance more comprehensively than modeling brain features based on individual lifestyle effects, supporting multivariate approaches in cognitive aging research.

Furthermore, the BF-Life_**multi**_ model showed an advantage over the fully data-driven models (without prior biological hypotheses) when both used the same feature-extraction procedure. In this procedure, features were selected or reduced from DISCOVERY before any model training. However, this advantage was conditional: the BF-Life_**multi**_ model did not outperform data-driven models when a more rigorous machine learning workflow was applied, in which PCA was performed separately within each training fold.

Importantly, the advantage of BF-Life_**multi**_ over both BF-Life_**single**_ and comparable data-driven models was sensitive to the granularity of the brain–lifestyle representation. When the cluster number was altered (*k* = 2–4), this advantage was not consistently observed (Suppl. Table 8 and Suppl. Fig. 4), suggesting that predictive performance may depend on how lifestyle-related variance is represented. In the present framework, the five-cluster solution appeared to capture a balance between aggregating shared lifestyle-related variance and avoiding excessive fragmentation.

Given the stronger prediction of figural memory, we next examined which brain features contributed most strongly to the BF-Life_**multi**_ predictions. SHAP feature importance analyses revealed that contributions to the predictions of the BF-Life_**multi**_ model primarily came from the salience/ventral attention, default mode, and somatomotor networks, as well as node-wise functional segregation within the visual network. These networks are broadly associated with attentional control, internally directed cognition, and visual processing^41–43^, functions relevant to figural memory performance.

At the feature level, important contributors included gray-matter morphology in salience/ventral attention and visual regions (e.g., right insula and right lingual gyrus), node-wise functional segregation within the visual network (e.g., left calcarine fissure), and microstructural integrity of projection fibers such as the posterior and superior corona radiata. Several of these regions have been reported to show structural associations with lifestyle factors, including smoking^44^, and are also implicated in visual processing. This overlap is consistent with their contribution to figural memory prediction in the present model. The high contribution of node-wise functional network segregation within the visual network may be viewed in the context of prior research on network dedifferentiation during aging and network reorganization in visual impairment^45,46^, where alterations in network segregation have been linked to differences in perceptual performance. Although the direction of this contribution remains unresolved, these findings suggest that visual-network segregation was one of the important contributors to the figural-memory model. In addition, the inclusion of projection-fiber integrity features suggests that white-matter microstructure may also be relevant to this domain, although the specific relationship remains to be clarified.

In contrast, BF-Life_**single**_ models reflected more specific aspects of lifestyle-related brain variability by emphasizing particular modalities or network subsets. BF-Life_**multi**_ integrates structural, functional, and microstructural features across multiple lifestyle domains, providing a more comprehensive representation than single-factor models. This multidomain perspective is consistent with previous work showing that combined lifestyle patterns are informative for cognitive aging^47^. Previous studies have also linked individual lifestyle factors to differences in brain structure and function^15–23,47^. Against this background, the present study examined whether brain features associated with multidomain lifestyle profiles also provide predictive information for cognitive performance. However, the modest prediction observed here indicates that lifestyle-related brain differences do not necessarily represent the brain differences most strongly related to cognitive performance.

The predictive advantage of the BF-Life_**multi**_ model was mainly observed for figural memory, whereas prediction was weaker or negative for other cognitive domains. This pattern may reflect the specific brain networks engaged in figural memory, which involve higher-order visual regions, whereas episodic memory relies more on medial temporal lobe structures^48^ and verbal abilities depend on widely distributed language-specific networks (e.g., prefrontal, temporal, and subcortical regions)^49^. The limited prediction of verbal tasks may also reflect the relative stability of these abilities across aging^50^, and they are strongly correlated with sociodemographic variables such as education and language exposure, which already encapsulate much of the neural information relevant to verbal performance^51^. By contrast, approaches such as connectome-based predictive modeling, which use target-dependent feature selection to target specific networks, have successfully predicted verbal abilities^31^, underscoring that prediction of different cognitive domains may benefit from tailoring feature selection and modeling approaches to the specific networks involved. However, the present study addressed a different question by selecting brain features based on lifestyle-profile differences, independent of cognitive outcomes. This design allowed us to test whether lifestyle-related brain variation carries predictive information for cognition without using the cognitive target itself to guide feature selection. The weaker prediction of verbal tasks therefore indicates that this lifestyle-profile-based feature set was not equally informative across cognitive domains.

Interestingly, the BF-Life_**multi**_ model also showed positive predictive performance for the global cognitive component. This may partly reflect that global cognitive functions rely on widespread and distributed neural networks, which tend to show relatively consistent large-scale organization compared with highly localized, domain-specific processes and are therefore more amenable to multimodal characterization^9^. Although the explained variance was modest, these results may reflect associations between lifestyle-related brain features and cognitive aging, especially for functions supported by distributed neural networks.

While the current study did not explicitly test aging frameworks, it exploratorily identified brain patterns—particularly in salience/ventral attention and visual networks—that may contribute to the prediction of specific cognitive domains. These brain patterns have not been directly examined in the context of established aging models in the current study, such as brain maintenance. This refers to the relative preservation of brain structure and function over the adult lifespan that is associated with better cognitive outcomes in aging^52^, and STAC–r, which suggests that older adults recruit additional neural circuits to compensate for age-related declines^53^. The present findings may nevertheless provide a basis for future studies examining how multidomain lifestyle-informed brain differences relate to domain-specific cognitive performance. Future studies could further investigate these links, for example, by incorporating task-based functional measures or additional lifestyle variables to enhance prediction.

Despite these brain-based findings, neuroimaging features alone accounted for only a small proportion of variance in cognitive performance. When demographic variables (age, sex, and education) were incorporated as additional predictors, predictive performance for figural memory improved substantially, consistent with prior findings that demographics capture a large share of explainable cognitive variance^7,11,54^. By contrast, the incremental contribution of brain features remained modest, and adding individual-level lifestyle variables did not yield further gains. This pattern suggests that variance associated with lifestyle may already be partially reflected in demographic and brain measures, leaving little additional predictive value for lifestyle variables themselves. This interpretation was also supported by the lifestyle-only sensitivity analysis, in which the original lifestyle variables by themselves showed weak prediction (Suppl. Table 10 and Suppl. Fig. 7). Nevertheless, a substantial proportion of variance in cognitive performance remained unexplained, indicating that future work may benefit from incorporating additional non-brain factors, such as socioeconomic status, physical health, cardiovascular risk, genetic predisposition, or social network characteristics.

In TEST_LONG_, predictive performance was markedly reduced. Neither BF-Life_**multi**_ nor the comparison models (BF-Life_**single**_ and data-driven models) showed meaningful predictive performance for figural memory at baseline or follow-up, even after incorporating demographic and lifestyle variables (Table 2). This consistent lack of performance across modeling approaches underscores the difficulty of predicting figural memory from brain features in this subgroup. Several factors may have contributed to this difference in prediction performance. Despite random assignment, TEST_LONG_ differed from DISCOVERY and TEST_CROSS_ in key characteristics, including education level and baseline figural memory performance (Table 1). In addition, the available training sample size may have been insufficient to learn patterns that generalize to a more distinct subgroup. Together, the TEST_LONG_ findings provide an important boundary condition for interpreting the present prediction results: predictive patterns observed cross-sectionally in DISCOVERY and TEST_CROSS_ did not translate into reliable prediction in TEST_LONG_. This may partly reflect heterogeneity within the sample and differences between the training and test samples, as well as the general challenge of predicting individual differences in cognitive performance from brain features, particularly across longitudinal assessments.

Furthermore, several methodological limitations warrant consideration. First, model performance was sensitive to clustering granularity, and separation between lifestyle subgroups was relatively weak (low silhouette scores; Suppl. Table 5), reflecting the difficulty of discretizing complex, multidimensional behaviors. Treating ordinal lifestyle variables as categorical inputs may also have reduced magnitude information. Some lifestyle indicators were also correlated and should therefore be interpreted as complementary indicators of broader behavioral domains rather than fully independent lifestyle dimensions (Suppl. Table 4). While clustering enabled the examination of broad lifestyle heterogeneity, future work could explore alternative methods and stability criteria to improve interpretability and robustness. Future work could also explore alternative feature-selection strategies that use the original lifestyle-variable structure without deriving discrete lifestyle subgroups.

Second, BF-Life_**multi**_ features were identified using exploratory between-group analyses without correction for multiple comparisons across all brain variables, with correction applied only across lifestyle subgroups. In addition, baseline health status or pre-existing disease may confound findings. Thus, results should be considered exploratory. In addition, since clustering and feature selection were performed once in DISCOVERY rather than repeated within each cross-validation fold, partial information leakage from validation folds into feature selection cannot be fully excluded. Cognitive outcomes were not used in these steps, so the potential leakage did not involve direct information about the prediction targets. The fixed feature set allowed the same lifestyle-informed brain representation to be evaluated across analyses.

Third, generalizability is constrained by lifestyle-variable selection, sample characteristics, and measurement design. The lifestyle profiles derived here reflect a selected set of behavioral and psychosocial domains and do not comprehensively capture all potentially relevant lifestyle or dementia-risk exposures, including domains such as diet and sleep. Furthermore, because the complete lifestyle-variable set required for clustering was not available for TEST_CROSS_, we could not directly evaluate whether this unseen dataset was comparable to DISCOVERY with respect to lifestyle profiles, and potential differences in lifestyle characteristics between the two subsets cannot be excluded. In addition, lifestyle behaviors were self-reported and subject to recall and social desirability biases, although this method is widely used in population neuroscience^26,55^.

Moreover, although longitudinal cognitive outcomes were examined, lifestyle and neuroimaging data were cross-sectional, limiting the ability to capture changes in these predictors and their relationship with cognitive change over time. Future research could further benefit from repeated behavioral and neural assessments over longer time spans to better characterize intraindividual differences in cognitive performance^56^. Finally, given the sociodemographic characteristics of the training sample, external validation in cohorts with broader educational, ethnic, and cultural representation is warranted before generalizing these findings^57^.

Despite these limitations, the current study suggests that brain features derived from overall lifestyle profiles provide modest domain-specific predictive information for cognitive performance in older adults, with positive predictive performance primarily observed for figural memory when using ENR. These lifestyle-related brain differences did not consistently surpass fully data-driven models and did not reliably predict longitudinal cognitive outcomes, but they provide a structured, lifestyle-informed representation of brain differences for combining lifestyle and neuroimaging data. Future work integrating larger and more diverse samples, repeated lifestyle and neuroimaging measures, and improved modeling of lifestyle heterogeneity may further clarify their relevance for cognitive aging.

## Methods

### Participants

The current study used data from the 1000BRAINS study^58^, nested within the population-based Heinz Nixdorf Recall Study^59^. We used lifestyle and psychosocial variables (hereafter referred to as lifestyle factors) from self-report questionnaires, along with baseline MRI and neuropsychological assessments at baseline and follow-up. We focused on participants aged ≥55 years (*n* = 969). Participants with DemTect scores in the impairment-indicative range (<8) or missing values were excluded (*n* = 23)^60,61^. Of this eligible sample, 859 participants had available structural magnetic resonance imaging (MRI), diffusion MRI, and resting-state functional MRI (rsfMRI). An additional 79 participants were excluded from further analyses, including 62 due to insufficient MRI data quality identified during initial quality control (see Section “Magnetic resonance imaging (MRI)”) and 17 due to additional quality issues (incomplete brain coverage, *n* = 1) or processing failures, including failures in diffusion model fitting (*n* = 2), spatial normalization (*n* = 9), and functional connectivity construction (*n* = 5). Additionally, participants missing more than three of the 13 cognitive assessments were excluded (*n* = 6), leaving 774 participants (mean age 67.0 ± 6.7 years, 46.1% females), of which 255 participants had both available lifestyle behavior data and follow-up cognitive measures (follow-up interval: 4.0 years).

Participants were divided into three non-overlapping subsets to allow model development and evaluation of cognitive prediction cross-sectionally and longitudinally: (1) a main dataset (DISCOVERY), used for lifestyle-profile derivation, feature selection and model training; (2) an unseen dataset (TEST_CROSS_), used to test the trained model in unseen participants cross-sectionally; and (3) a second unseen dataset (TEST_LONG_), used to test model performance in second-visit data.

In detail, (1) in DISCOVERY lifestyle-profiles were derived with cross-validation used for internal model evaluation (*n* = 453; mean age 67.7 ± 6.6 years; 47.0% females); (2) TEST_CROSS_ consisted of participants with baseline MRI and cognitive assessments who were held out from lifestyle-profile derivation because the complete lifestyle-variable set required for clustering was not available for this test subset, as several of these variables had not been collected under the participants’ original study protocol (*n* = 211; mean age 66.3 ± 7.1 years; 47.4% females); and (3) TEST_LONG_ participants (*n* = 110; mean age 65.3 ± 6.2 years; 40.0% females; follow-up interval: 4.0 years) were randomly selected a priori from the 255 participants with available lifestyle behavior data and follow-up cognitive measures, providing an independent evaluation set while retaining sufficient sample size for model training. The remaining eligible participants (*n* = 145) were retained in the DISCOVERY dataset.

All participants provided written informed consent. The 1000BRAINS protocol was approved by the Ethics Committee of the University of Duisburg-Essen, Germany, and conducted in accordance with the Declaration of Helsinki.

Lifestyle and psychosocial data used here were retrieved from the database of the third examination of the Heinz Nixdorf Recall study (10-year follow-up)^58,59^. For the present analysis, we focused on eight lifestyle and psychosocial indicators spanning smoking, alcohol consumption, physical activity/mobility, and the broader social engagement domain. These domains were selected to capture distinct but partly related aspects of everyday lifestyle and psychosocial behavior that could be operationalized consistently within the present analytical framework, with prior evidence supporting their relevance to brain and cognitive aging as outlined in the Introduction^16–23^. Specifically, we considered: (1) smoking [packyears of smoking = years of smoking × self-reported number of cigarettes smoked per day (CPD)^20^], (2) alcohol consumption [total monthly consumption of pure alcohol (g/month)], (3) physical activity [sum of metabolic equivalents of task (MET) values assigned to each of the activities (sports/daily physical activities) participants engaged in multiplied by duration in hours (per month)]^62^, (4) flights of stairs [number of flights of stairs participants climbed daily^63^], (5) count of sporty activities [number of different types of sports-related activities (e.g., running) that participants reported engaging in during the previous month], (6) count of physical activities [number of different types of physical activities (e.g., gardening) that participants reported engaging in during the previous month], (7) social integration, and (8) count of visits [sum of visits from children, relatives, and friends within one month]. Social integration was assessed following the adapted Social Integration Index used by ref. ^25^, based on the established epidemiological index described by ref. ^64^, and comprised three domains: marital status, close ties, and membership in organizations. Visit count was not included in this index and was entered separately as an indicator of visit frequency. We replaced missing values with the mean values from the first two examinations^65^. Thus, MET reflected intensity- and duration-weighted activity, whereas stairs and activity counts captured specific mobility behaviors and activity diversity; similarly, visit count was entered separately from social integration to capture visit frequency.

Variables were transformed into ordinal categories for subsequent clustering analysis. Smoking (SMO) was coded as never, former, or current, while alcohol consumption (ALC) was coded as never, former, current non-spirits, or current spirits drinkers according to longitudinal self-reports. The remaining six variables—physical activity (PA), social integration (SI), flights of stairs climbed, count of visits, count of sporty activities, and count of physical activities—were divided into quartiles (Q1–Q4). All variables were coded such that higher values reflected higher risk for brain health (e.g., heavier smoking, higher alcohol intake, lower physical activity).

All participants underwent a comprehensive neuropsychological assessment during the 1000BRAINS data collection, which covered a broad range of cognitive functions. Thirteen cognitive tests were included in the present study, covering cognitive status (DemTect, indicating overall cognitive performance), language, memory, working memory, verbal fluency, and executive control. The prediction targets were labeled by cognitive domain for readability; the corresponding test names, references, cognitive domains, and scoring definitions are provided in Suppl. Table 1^11,58^. Participants missing more than three cognitive tests were excluded (*n* = 6). For tests with fewer than 3% missing data, missing scores were replaced with the median value of the four participants nearest in age (the two closest younger and the two closest older than the individual)^66^.

In addition to domain-specific analyses, we included a global cognitive component score based on previous 1000BRAINS work^7,11^, which was constructed using a partly overlapping but not identical subset of neuropsychological tests compared to the 13 cognitive assessments used in the present study, to ensure comparability with prior findings and to assess whether prediction performance generalizes beyond individual domains.

### Magnetic resonance imaging (MRI)

Magnetic resonance imaging (MRI) was carried out on a 3T Siemens Tim-TRIO MR scanner with a 32-channel head coil (Erlangen, Germany) during the 1000BRAINS data collection, on the same day as the neuropsychological examination. Out of the full study protocol^58^, T1-weighted, diffusion and resting-state functional MRI were included in the present analysis with the following sequence details. High-resolution T1-weighted imaging data were acquired with 176 slices, slice thickness = 1 mm, repetition time (TR) = 2250 ms, echo time (TE) = 3.03 ms, field of view (FoV) = 256 × 256 mm^2^, flip angle = 9°, voxel resolution 1 × 1 × 1 mm^3^. Diffusion-weighted imaging (dMRI) data were acquired using a twice refocused spin-echo diffusion-weighted EPI sequence with the following two parameter sets (each with FoV = 216 × 216 × 132 mm^3^, voxel resolution = 2.4 × 2.4 × 2.4 mm^3^, and 55 interleaved transversal slices without inter-slice gap): (i) 120-direction subset: 120 diffusion-weighted images (*b* = 2700 s/mm^2^), 13 *b*0-images, TR = 8 s, TE = 112 ms; (ii) 60-direction subset: 60 diffusion-weighted images (*b* = 1000 s/mm^2^), 7 *b*0-images, TR = 6.3 s, TE = 81 ms. Resting-state functional MRI (rsfMRI) data were acquired using a blood-oxygen-level-dependent (BOLD) gradient-EPI sequence with 36 transversal slices (slice thickness = 3.1 mm, TR = 2200 ms, TE = 30 ms, FoV = 200 × 200 mm^2^, voxel resolution 3.1 × 3.1 × 3.1 mm^3^), lasting ~11 min and producing 300 volumes. During scanning, participants were instructed to keep their eyes closed, remain relaxed, and avoid falling asleep.

T1-weighted data preprocessing is detailed in the previous 1000BRAINS work^7^. We adapted FreeSurfer’s ‘recon-all’ pipeline (version 7.1.0; https://surfer.nmr.mgh.harvard.edu/) to include the 400-node Schaefer parcellation^67^, which was transformed into individual subject space using *mris_ca_label*. Morphological values for each node were then extracted with *mris_anatomical_stats*.

The dMRI data were preprocessed, including correction for eddy currents, head motion artifacts, and slice-wise signal dropouts^68^, using FSL (version 6.0; https://fsl.fmrib.ox.ac.uk/fsl/). Multi-shell data (*b*1000 and *b*2700) were then aligned to the *b*0 image of the *b*1000 shell using linear registration (*FLIRT*) and merged into a single dataset. Diffusion tensors were fitted to the *b*1000 shell using *dtifit* to generate fractional anisotropy (FA), mean diffusivity (MD) [(λ1 + λ2 + λ3)/3], radial diffusivity (RD) [(λ2 + λ3)/2], and axial diffusivity (AD) (λ1) maps. For NODDI modeling, we used the Microstructure Diffusion Toolbox (MDT, https://mdt-toolbox.readthedocs.io) based on combined multi-shell data, generating voxel-wise maps of intracellular volume fraction (ICVF) and orientation dispersion index (ODI). Diffusion MRI quality control metrics were obtained from the EDDY QC framework (Study-Wise Quality Assessment, SQUAD)^69^. Among these, the contrast-to-noise ratio (CNR) was used for quality assessment, and participants with CNR values exceeding ±2 standard deviations from the group mean were excluded to reduce the impact of low-quality data (*n* = 15).

For rsfMRI preprocessing, we followed the established pipeline from previous 1000BRAINS work^60^, including volume removal, motion correction, ICA-based Automatic Removal Of Motion Artifacts (ICA-AROMA)^70^, filtering, and normalization to MNI152 space. Participants were excluded based on the following quality control criteria: (1) missing AROMA outputs (*n* = 19); (2) failures in CAT12 preprocessing (i.e., missing scans in the homogeneity check output; *n* = 5); (3) outliers identified by CAT12 sample homogeneity checks (>2 standard deviations from the group mean; *n* = 8); (4) inconsistent numbers of functional volumes (*n* = 9); and (5) excessive motion-related artifacts identified using DVARS, defined as >10% frame dropout across 300 volumes (*n* = 6)^7,60^.

### Brain features

For brain structure, gray-matter volume (GMV), cortical thickness (CT), and surface area (SA) were extracted for 400 nodes (3 × 400 features) of the Schaefer 400 parcellation^67^ using *mris_anatomical_stats*, and summarized using the *aparcstats2table* program in FreeSurfer.

Individual DTI and NODDI metrics of all subjects were nonlinearly transformed into MNI space using ANTs. Within white-matter regions of interest as derived from the ICBM-DTI-81 white matter label atlas^71^, which comprises 48 labels, we extracted the average values of FA, MD, RD, AD, ICVF, and ODI for each participant using *fslstats* tool. These white matter region-wise DTI and NODDI metrics (resulting in 6 × 48 features) were then used as input features for subsequent analyses.

To capture large-scale functional brain organization across the cerebrum, cerebellum, and subcortical regions^72^, we constructed FC matrices using a combined parcellation consisting of 400 cortical parcels^67^, 14 subcortical regions^73^, and 7 cerebellar regions^74^, resulting in 421 nodes in total. RsfMRI time series were averaged within each node, and pairwise Pearson correlations were computed between nodes to obtain FC values per each node pair. This resulted in a symmetric connectivity matrix of 421 nodes × 421 nodes. Non-significant connections were excluded by permutation testing, and Fisher’s *r*-to-*z* transformation was applied^60,75,76^. Absolute values were used to prevent positive and negative weights from offsetting each other when estimating connection strength. To avoid distortions caused by differences in network density and overall FC, no additional thresholding was performed to sparsify the network^60,75,77^. From the absolute FC matrices, three graph-theoretical parameters were derived node- and network-wise using custom R scripts^75^, following previously established definitions^4^: (i) within-network FC strength, (ii) between-network FC strength, and (iii) a segregation score that quantifies the relative balance of connectivity within networks versus connectivity between networks. The 421 nodes each yielded a segregation value, producing 421 node-wise features. In addition, network-wise metrics were extracted. With the regions of the Schaefer 400 parcellation being assigned to 9 large functional brain networks (i.e., visual, somatomotor, dorsal and salience/ventral attention, limbic, frontoparietal control, default mode, subcortical, and cerebellar networks), a total of 27 network-wise metrics (9 networks × 3 FC graph-theoretical parameters) could be derived.

Together with structural MRI and dMRI features, this resulted in a total of 1936 whole-brain features used for subsequent analyses.

### Machine learning framework for cognitive function prediction

**Fig. 6:**
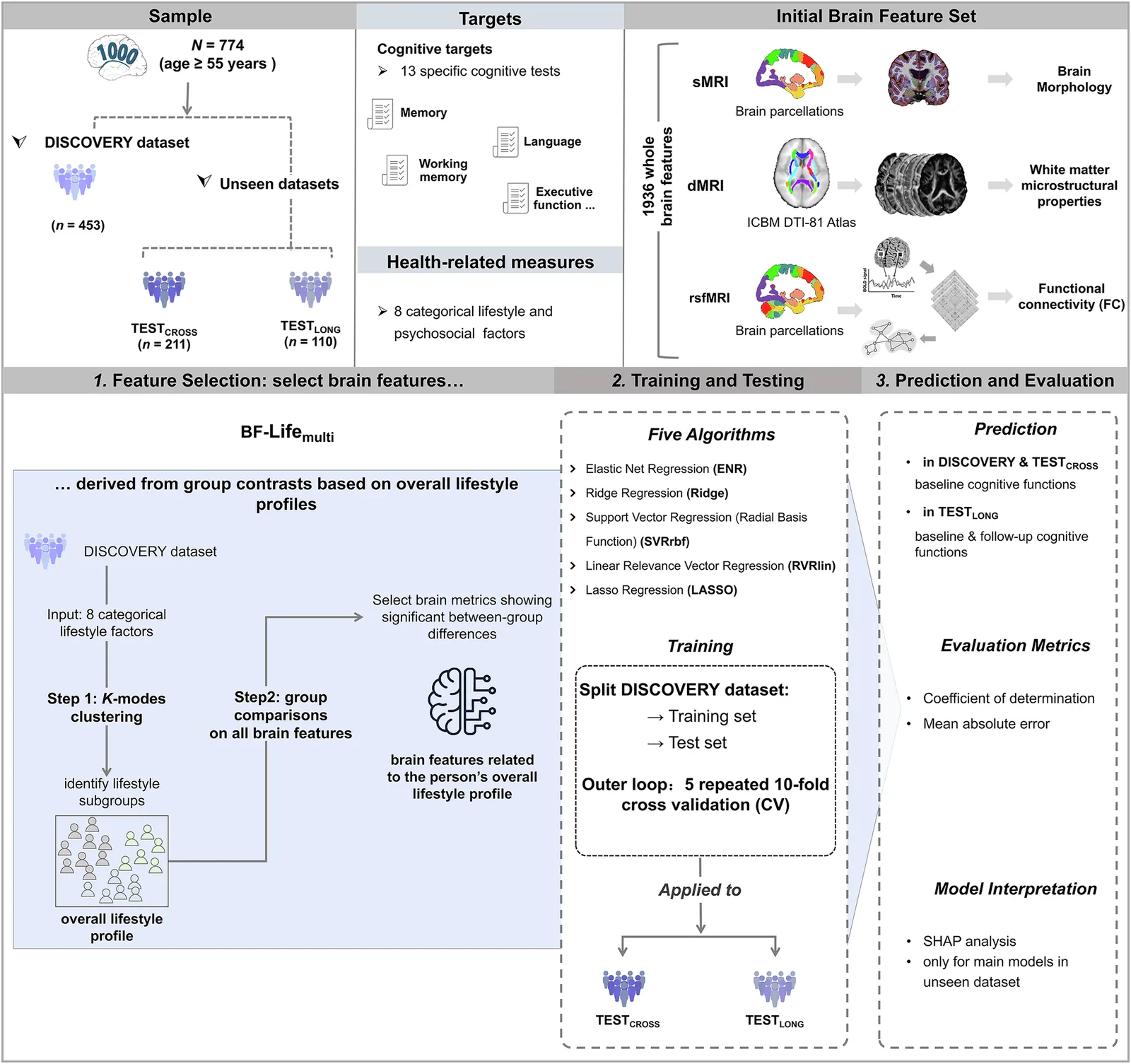
Overview of the workflow. Samples were divided into a DISCOVERY dataset and two unseen TEST sets. The prediction targets consisted of 13 cognitive tests. From 1936 brain imaging features, feature extraction was performed to construct the main model with brain features derived from overall lifestyle profiles, which were identified through clustering and between-group comparisons (BF-Life_**multi**_). Models were trained using five repeated nested cross-validation and evaluated using *R²* and MAE. Model interpretation was performed using SHAP analysis on the unseen dataset.

This study aimed to evaluate if prediction of cognitive outcome from brain parameters would benefit from a model setup which is based on dedicated brain features associated with a person’s overall lifestyle profile. Thus, in our main analyses, we developed a model using brain features resulting from analyses investigating differences between groups of participants with different lifestyle profiles (BF-Life_**multi**_). We compared the BF-Life_**multi**_ model against (i) a model using features related to a single lifestyle factor (BF-Life_**single**_), (ii) a fully data-driven model, trained on principal components from the total amount of 1936 brain features (BF_data_), and (iii) a model that incorporated demographic and lifestyle data in addition to the brain features into the BF-Life_**multi**_ model. A schematic overview of the machine learning workflow is shown in Fig. 6. Details are described below.

The BF-Life_**multi**_ model was developed in four main steps: lifestyle clustering, identification of lifestyle-related brain features, model training using multiple algorithms within a nested cross-validation framework, model evaluation, and model interpretation using SHAP (SHapley Additive exPlanations)^78^.

The lifestyle clustering analysis aimed to identify groups of participants with similar lifestyle behavior within groups and differing behavior between groups. Therefore, *K*-modes clustering was used to group participants in the DISCOVERY dataset based on their individual profiles. Each DISCOVERY participant was represented by the eight lifestyle factors described in section “Participants”, forming a participant-by-lifestyle-variable matrix. These ordinal lifestyle variables were treated as categorical inputs in *K*-modes clustering (version 0.12.2) to reduce sensitivity to extreme values. This categorization was used to emphasize relative lifestyle levels in this relatively healthy sample, and *K*-modes was chosen because it is designed for categorical variables and supports a person-centered derivation of interpretable lifestyle profiles. Brain, cognitive, and demographic variables were not included in the clustering procedure. In detail, the algorithm was run with (i) 1000 random initializations for robust centroid selection; (ii) participants assigned to the nearest cluster using Hamming distance; (iii) cluster modes updated; and (iv) steps 2–3 repeated until assignments stabilized or 300 iterations were reached. The optimal number of clusters (*k*) was chosen by balancing statistical criteria and interpretability, using *SW/SB* ratio (within-cluster to between-cluster standard deviation ratio), *R*-squared, Silhouette score, and Davies–Bouldin index (DBI)^79^, where lower *SW/SB* and DBI, and higher *R*-squared and Silhouette values indicate better clustering^80^. We tested *k* = 2–10 and evaluated the clustering solutions using these indices. Because such indices often favor simpler solutions (e.g., lower *k* values), the final *k* = 5 solution was selected by balancing statistical performance with biological interpretability^81^.

To identify brain phenotypes related to different lifestyle behavior, feature selection was performed by identifying brain features that differed between our 5 lifestyle subgroups. In detail, feature selection was performed by comparing all 1936 brain features between the identified *k* = 5 lifestyle subgroups. Cognitive outcomes were not used during this feature-selection step. Due to the very large number of brain features relative to our sample size, univariate ANCOVAs were used for feature selection, adjusting for age, sex, education, and intracranial volume (for morphometric measures), to reduce demographic and head-size confounding during feature selection. This adjustment was used only to identify lifestyle-subgroup-related brain features; the selected brain-feature values were not residualized for these covariates before entering the prediction models. Brain metrics showing significant differences between any subgroup pair were retained. Significant group differences were identified at *p* < 0.05 with Bonferroni-corrected post hoc tests between subgroups (*q* < 0.05). Non-parametric Quade ANCOVA^82^ was applied when variance assumptions were violated. The resulting BF-Lif**e**_**multi**_ feature set was held fixed during cross-validation and subsequent testing to maintain a consistent lifestyle-subgroup definition and corresponding brain-feature representation across analyses. No correction for multiple comparisons across neuroimaging metrics was applied, allowing richer input for the exploratory identification of potentially lifestyle-relevant features; accordingly, the selected features should be interpreted as an exploratory input set for prediction.

The primary goal of the current study was to test whether the BF-Life_**multi**_ feature set provides helpful information for cognitive prediction. However, we assessed the robustness of prediction patterns across different modeling assumptions by applying five regression-based algorithms commonly used in neuroimaging analyses: elastic net regression (ENR), ridge regression, support vector regression with the radial basis function kernel (SVRrbf), relevance vector regression with linear kernel (RVRlin), and least absolute shrinkage and selection operator (LASSO) regression. Model fitting used a nested cross-validation (CV) framework within DISCOVERY, with an outer 5-repetitions × 10-fold CV for training/test splits and an inner 10-fold CV for hyperparameter tuning. For ENR, the regularization strength (α) was tuned over logarithmically spaced values ranging from 10⁻⁴ to 10³, and the L1 ratio varied between 0.1 and 0.9. For ridge regression, the regularization parameter α was tuned over the same logarithmic range. For SVRrbf, the regularization parameter *C* varied between 10⁻³ and 10³, and the kernel width parameter γ was set to either scale or auto. For LASSO, the regularization parameter α was tuned over logarithmically spaced values from 10⁻¹ to 10⁵. For RVRlin, the hyperparameters *α* and *β* were tuned over values spanning multiple orders of magnitude (10⁻⁶, 1, and 10³). In all cases, hyperparameter selection was performed exclusively within the inner cross-validation loop. To evaluate generalization beyond model development, models trained in DISCOVERY were applied to the unseen TEST_CROSS_ and TEST_LONG_ subsets. Features were standardized (mean = 0, standard deviation = 1) within each cross-validation split, using parameters estimated from the training fold and then applied to the corresponding test fold and unseen datasets. All predictions were based on baseline MRI data.

Performance of all prediction models was evaluated using the coefficient of determination (*R²*), indicating the explained variance, and the mean absolute error (*MAE*). For example, an *MAE* of 1 point means that predictions deviate from observed scores by an average of ±1 (e.g., seconds or correct words). Feature importance was assessed using SHAP analysis for the BF-Life_**multi**_ model in the unseen datasets. To avoid interpreting feature contributions from models without meaningful predictive performance, SHAP interpretation was restricted to the algorithm and unseen dataset showing positive and comparatively stable prediction. Linear explainers were used for ENR, ridge, and LASSO, and kernel explainers for RVRlin and SVRrbf. For each feature, contributions were calculated as the average absolute SHAP value across the 50 cross-validation iterations (5 repeats × 10 folds).

For region-wise features, each feature label includes the functional network (e.g., SAN), the Schaefer parcellation region (e.g., FrOperIns_2), and the corresponding cytoarchitectonic area from the Juelich brain atlas (e.g., Id6)^83^, allowing precise anatomical interpretation.

Given the high dimensionality of the voxel- and region-wise features, we improved interpretability by aggregating SHAP values into broader, biologically meaningful categories (e.g., GMV in the visual network, FA in the association fiber tract), defined according to established brain atlases or tractographies. Category-level importance was then obtained by summing SHAP values within each category and ranking the results. All analyses were conducted in Python (v3.10.9) with scikit-learn (v1.1.2).

For comparison, three comparison models were constructed alongside the BF-Life_**multi**_ model (see below). Among the five algorithms evaluated within the BF-Life_**multi**_ model, one algorithm was selected for focused model comparisons based on its overall predictive performance across cognitive targets in DISCOVERY, as quantified by *R²*. To ensure comparability, all comparison models were implemented using this selected algorithm and the same hyperparameter-tuning procedure.

To assess whether brain features derived from overall lifestyle profiles provide predictive information beyond those related to individual lifestyle factors, we constructed four models based on brain features reflecting the effects of a single lifestyle-related factor (BF-Life_**single**_): smoking (SMO), alcohol consumption (ALC), physical activity (PA), and social integration (SI). For each BF-Life_**single**_ model, participants were grouped separately according to one corresponding lifestyle factor. Lifestyle-specific brain features were then identified using the same ANCOVA-based procedure as in BF-Life_**multi**_. In these analyses, age, sex, education, intracranial volume (for morphometric measures), and the remaining three lifestyle-related factors were included as covariates to isolate the effect of the factor of interest. The resulting brain features were used to construct separate machine learning models. SHAP analysis was applied to all BF-Life_**single**_ models using the same settings as for the BF-Life_**multi**_ model.

To compare the BF-Life_**multi**_ model against fully data-driven models, we applied principal component analysis (PCA)-based dimensionality reduction to all 1,936 brain features (Suppl. Table 2). PCA was applied either to DISCOVERY or to the training set within each cross-validation fold only. For each case, the number of components was defined in two ways: either to explain 80% of the total variance or to match the number of features used in the BF-Life_**multi**_ model. This resulted in four feature sets: BF_data_-Var and BF_data_-Match (PCA applied to DISCOVERY), and BF_data_-Var[Train] and BF_data_-Match[Train] (PCA applied to the training set). All hyperparameters were kept identical to those in the BF-Life_**multi**_ model to ensure comparability, and analyses focused on the algorithms and cognitive targets that showed the highest predictive performance in that model.

We extended the BF-Life_**multi**_ model by incorporating demographic variables (i.e., age, sex, and education; BF-Life_**multi**_ +dem), as well as the original eight continuous lifestyle variables (BF-Life_**multi**_ +dem+life), to assess the added predictive value of these non-brain variables beyond the selected, non-residualized brain features. Although the same eight lifestyle variables, recoded as categorical variables, were initially used to derive the lifestyle subgroups and thereby indirectly informed brain-feature selection, their original continuous values were not included as predictors in the BF-Life_**multi**_ model.

As sensitivity analyses, we assessed three alternative model setups using the algorithm that showed the most stable predictive performance in the main analysis, with the same hyperparameter search settings (see section “Machine learning framework for cognitive function prediction”). First, for cognitive targets with positive predictive performance in the main analysis (*R²* > 0), we evaluated the impact of alternative clustering solutions for deriving overall lifestyle profiles (*K*-modes with *k* = 2–4). For the cognitive outcome with the highest predictive performance in the main analysis, we further conducted SHAP analyses to characterize feature contributions. Second, we implemented lifestyle-only models using the original eight continuous lifestyle variables as predictors to provide a reference for the predictive performance of lifestyle variables themselves. These analyses were restricted to datasets with complete lifestyle data and were treated as sensitivity analyses because the main focus was lifestyle-informed brain-feature selection. Third, sensitivity toward specific cognitive domains was examined by extending the predictions to a global cognitive component score as a supplementary target.

### Statistical analyses

Basic characteristics of the total sample, DISCOVERY, and unseen datasets are summarized in Table 1. Differences between the DISCOVERY and unseen datasets were assessed using ANOVA for normally distributed variables, Chi-square tests for sex, and Mann–Whitney *U*-tests for non-normally distributed variables. Pearson correlations among the eight lifestyle variables were calculated to characterize overlap between lifestyle indicators and are reported in the Suppl. Table 4. For post hoc characterization, lifestyle subgroups were compared in terms of demographic, lifestyle, and cognitive variables using Chi-square tests, ANOVA, and Quade's ANOVA, where appropriate. To describe broader brain patterns after subgroup assignment, BF-Life_**multi**_ features were averaged within each feature category (e.g., GMV and CT) and analyzed using ANOVA. All analyses were performed in IBM SPSS Statistics 29.0.

## Supplementary information

Supplementary information

## Data Availability

This study used data from the 1000BRAINS study, a population-based human neuroimaging cohort. Access to raw neuroimaging and phenotypic data is restricted due to ethical and data protection regulations. Data are available to qualified researchers upon reasonable request to the 1000BRAINS study organizers. Derived imaging measures that support the findings of this study are available from the corresponding author upon reasonable request.

## Code availability

The custom code used for data preprocessing, lifestyle clustering, brain-feature construction, predictive modeling, model comparison, and sensitivity analyses is available from the corresponding author upon reasonable request.
