# Supplementary material for: Predictive performance of lifestyle-informed multimodal brain features for cognition in healthy older adults
Source: NPJ Aging. 2026 Nov 14;12(1):159. doi: 10.1038/s41514-026-00558-4 (PMC13769762; doi:10.1038/s41514-026-00558-4)
Supplement: Supplementary file 1 [file 41514_2026_558_MOESM1_ESM.pdf]

Supplementary Materials

Content List

Abbreviations List

Table List

Table S1 Definition of cognitive prediction targets and neuropsychological measurements.
Table S2 Summary of the machine learning models.
Table S3 Baseline and follow-up neuropsychological scores of participants in the TESTLONG dataset.
Table S4 Pearson correlation matrix among lifestyle features.
Table S5 Optimal *k* value in *K*-modes clustering.
Table S6 Characteristics of lifestyle subgroups.
Table S7 Performance of the BF-Life$_{\mathbf{multi}}$ model in predicting figural, episodic, and long-term memory.
Table S8 Top 30% features in the BF-Life$_{\mathbf{multi}}$ model in predicting figural memory using ENR and SHAP analysis.
Table S9 Sensitivity analysis: Performance of the BF-Life$_{\mathbf{multi}}$ model using ENR under alternative clustering solutions
Table S10 Sensitivity analysis: Performance of lifestyle-only reference models in predicting figural memory.
Table S11 Sensitivity analysis: Performance of the 11 models using ENR on the global cognitive component.

Figures List

Figure S1 Lifestyle (A) and brain (B) profiles of each lifestyle subgroup.
Figure S2 Cognitive profiles of each lifestyle subgroup.
Figure S3 Feature selection frequency in the BF-Life$_{\mathbf{multi}}$ and BF-Life$_{\mathbf{single}}$ models, grouped by feature category.
Figure S4 Predictive performance of various models for figural memory using ENR.
Figure S5 Importance of feature categories in nine models predicting figural memory using ENR in the TEST$_{\mathrm{CROSS}}$ dataset.
Figure S6 Feature importance in predicting figural memory using single-factor models and SHAP analysis with ENR.
Figure S7 Predictive performance of lifestyle-only reference models using five machine learning algorithms.
Figure S8 Predictive performance of various models for the global cognitive component using ENR.

eReferences

Abbreviations List

Models and algorithms

ENR: Elastic Net Regression
Ridge: Ridge regression
Lasso: Least Absolute Shrinkage and Selection Operator regression
SVRrbf: Support vector regression with radial basis function kernel
RVRlin: Relevance vector regression with linear kernel
BF-Life$_{\mathbf{multi}}$, brain features derived from group contrasts based on multiple lifestyle profiles
BF-Life$_{\mathbf{single}}$, brain features derived from group contrasts based on a single lifestyle factor
SMO: BF-Life$_{\mathbf{single}}$ models based on smoking
ALC: BF-Life$_{\mathbf{single}}$ models based on alcohol consumption
PA: BF-Life$_{\mathbf{single}}$ models based on physical activity
SI: BF-Life$_{\mathbf{single}}$ models based on social integration
PCA: Principal component analysis
+dem: includes demographic data
+dem+life: includes demographic and lifestyle data
k: Cluster number

Brain networks

VIN: Visual network
SMN: Somatomotor network
DAN: Dorsal attention network
SAN: Salience/ventral attention network
LIN: Limbic network
FPN: Frontoparietal control network
DMN: Default mode network
Sub: Subcortical network
Cereb: Cerebellar network

Brain features in the gray matter

GMV: Gray matter volume
CT: Cortical thickness
SA: Surface area
FC: Functional connectivity
net FC: Network-wise FC-related metrics
node FC: Node-wise functional segregation

Brain features in the white matter

FA: Fractional anisotropy
MD: Mean diffusivity
AD: Axial diffusivity
RD: Radial diffusivity
ICVF: Intracellular volume fraction
ODI: Orientation dispersion index

White matter fiber tracts

Asso: Association fiber tract

Proj: Projection fiber tract

BS: Brainstem fiber tract

Comm: Commissural fiber tract

Other variables

EDU: Education level

Table S1 Definition of cognitive prediction targets and neuropsychological measurements.

Prediction target label	Test (References)	Cognitive domain	Scoring definition
Cognitive status	DemTect [1]	Cognitive functioning	The sum of the scores from a digit span memory test, a number conversion task, and a verbal fluency task (sum score).
Language comprehension	Wortschatztest (Word-hoard-test) [2]	General language comprehension, Vocabulary	Total number of target words correctly identified among distractors (sum score).
Nomination	Boston Naming Test (from CERAD; short form) [3]	Nomination	The number of correct answers on an object-naming task (using frequent, medium-frequent, and rare items) (sum score).
Figural memory	Benton-Test [4]	Visual / figural memory	Total number of correctly free recalled geometric figures from 20 previously presented figures (sum score).
Verbal WM	Zahlennachsprechen (from Nürnberger Alters-Inventar) [5]	Verbal working memory	Total number of correctly recalled digits repeated in a backward sequence (sum score).
Phonemic fluency	Regensburger Wortflüssigkeitstest [6]	Phonemic fluency	Total number of produced words beginning with a given letter B in 2 minutes (sum score).
Phonemic switch		Phonemic switch	Total number of produced words beginning with a given letter G-R in 2 mins (sum score).
Semantic fluency		Semantic verbal fluency	Total number of produced words in the category “Berufe (job)” in 2 mins (sum score).
Semantic switch		Semantic verbal switch	Total number of produced words in the category “sports-fruits” in 2 mins (sum score).
Visuospatial WM	Corsi block tapping task (CBT) [7]	Visual-spatial working memory	The number of correct answers on a backward block sequence recall task (with gradually increasing sequence length) (sum score).
Episodic memory	Bielefelder Kategorielle Wortlisten (BKW) [8]	Verbal learning / episodic memory	The average of the relative proportions of reproduced free recalls across five runs.
Long-term memory		Verbal delayed recall / long-term memory	Total number of correctly free recalled words from a list of 15 words after approx. 30 mins (sum score).
Executive control	Trail making test (TMT) A and B (taken from CERAD-Plus) [3]	Cognitive flexibility, concept shifting	Ratio of completion time (sec.) for TMT Part B to TMT Part A; lower values indicate better executive control/cognitive flexibility.

Note: Prediction target labels are used in the main text and figures for readability; the corresponding neuropsychological test names and scoring definitions are provided here. Further descriptions are also found in Caspers et al., 2014 [9]. Abbreviations: WM, working memory

Table S2 Summary of the machine learning models.

Models	Feature count	Strategy	Description of feature selection
**BF-Life$_{multi}$ models**			
k = 5*	97		Step 1: clustering lifestyle subgroups using eight ordinal lifestyle variables as input in the DISCOVERY dataset. Step 2: performing univariate analyses on 1,936 brain features to identify intergroup differences among identified lifestyle subgroups (DISCOVERY dataset).
k = 2	151		
k = 3	102		
k = 4	42		
**Incorporating non-brain data**			
BF-Life$_{multi}$ +dem: k = 5	100	Lifestyle-informed	Add demographics data on top of BF-Life$_{multi}$ features
BF-Life$_{multi}$ +dem+life: k = 5	108		Add continuous lifestyle variables on top of BF-Life$_{multi}$+dem features
**BF-Life$_{single}$ models**			
SMO	117		Performing univariate analyses on 1,936 brain features separately for each single lifestyle factor to identify intergroup differences among behavioral subgroups (DISCOVERY dataset).
ALC	54		
PA	80		
SI	81		
**Data-driven models**			
BF$_{data}$-Var	166-167	Data-driven reduction (target-independent)	Applying PCA to all 1,936 brain features, with component number set either to explain 80% variance or to match the BF-Life$_{multi}$ feature size (DISCOVERY dataset).
BF$_{data}$-Match	97		
BF$_{data}$-Var(Train)	155-156		Applying PCA to all 1,936 brain features, with component number set either to explain 80% variance or to match the BF-Life$_{multi}$ feature size (training set).
BF$_{data}$-Match(Train)	97		

Abbreviations: BF-Life$_{multi}$, brain features derived from group contrasts based on multiple lifestyle profiles; k, cluster number; BF-Life$_{single}$, brain features derived from group contrasts based on a single lifestyle factor; SMO / ALC / PA / SI: BF-Life$_{single}$ models based on smoking, alcohol consumption, physical activity, and social integration, respectively; BF$_{data}$, data-driven PCA brain features; Var = 80% variance explained; Match = matched feature number; [Train] = PCA applied in training set exclusively; +dem, includes demographic data; +dem+life, includes demographic and lifestyle data

Table S3 Baseline and follow-up neuropsychological scores of participants in the $\text{TEST}_{\text{LONG}}$ dataset.

	$\text{TEST}_{\text{LONG}}$ (n = 110)	
	Baseline	Follow-up
Age	65.3 (6.2)	69.35 (6.0)
Cognitive status	14.93 (2.47)	14.95 (2.43)
Language comprehension	31.36 (4.02)	31.18 (3.91)
Nomination	14.59 (0.69)	14.5 (0.85)
Figural memory	9.94 (3.70)	9.96 (3.86)
Verbal WM	4.65 (0.93)	4.61 (1.06)
Phonemic fluency	18.58 (6.26)	18.86 (6.50)
Phonemic switch	19.05 (5.99)	18.5 (5.80)
Semantic fluency	23.61 (7.06)	24.15 (7.42)
Semantic switch	19.86 (4.57)	20.33 (4.73)
Visuospatial WM	4.78 (1.10)	4.71 (1.10)
Episodic memory	62.49 (11.90)	68.99 (10.26)
Long-term memory	10.95 (2.80)	12.03 (2.39)
Executive control	2.38 (0.74)	2.38 (0.76)

Note: The mean value and standard deviation are represented as mean (*SD*). Abbreviations: WM, working memory

Table S4 Pearson correlation matrix among lifestyle features.

	Packyears	Alcohol	Physical activity	Stairs	Social integration	Visit_count	Sporty_count	Physical_count
Packyears	--							
Alcohol	0.05	--						
Physical activity	0.00	-0.03	--					
Stairs	-0.02	0.04	0.08	--				
Social integration	-0.09*	0.09*	0.00	0.02	--			
Visit_count	-0.02	0.04	-0.02	-0.01	0.71***	--		
Sporty_count	-0.15***	0.00	0.31***	0.12**	0.04	0.05	--	
Physical_count	-0.07	0.01	0.10*	0.06	0.07	0.01	0.12**	--

Note. Values are unadjusted Pearson correlation coefficients. Significance stars indicate two-sided Pearson correlation tests: *, $p < .05$; **, $p < .01$; ***, $p < .001$. The lower triangle is shown; diagonal cells are marked with --. Pairwise $N = 563$ for all correlations. Abbreviations: Packyears, packyears of smoking; Alcohol, alcohol consumption; Physical activity, measured by metabolic equivalent of the task; Stairs, flights of stairs climbed; Visit_count, count of visits from children, relatives, and friends; Sporty_count, count of sporty activities; Physical_count, count of physical activities

Table S5 Optimal *k* value in *K*-Modes clustering.

**Number of clusters (*k*)**	***SW*/*SB* ratio**	***R*-Squared**	**Silhouette Score**	**Davies-Bouldin Index (DBI)**
2	**0.029**	**0.972**	**0.111**	**2.438**
3	0.038	0.963	*0.107*	4.682
4	0.037	0.964	0.105	3.577
5	*0.035*	*0.967*	*0.107*	3.117
6	0.038	0.964	0.090	*3.065*
7	0.040	0.962	0.090	3.259
8	0.045	0.957	0.089	3.641
9	0.046	0.956	0.091	3.303
10	0.046	0.956	0.094	3.462

Note: A lower *SW*/*SB* ratio and DBI score indicate better clustering performance, while higher *R*-squared and Silhouette scores suggest better cluster separation. Bold indicates the optimal solution; italics indicate the suboptimal solution. Although *k*=2 yields the best performance across all metrics, we selected *k*=5 as the optimal number of clusters. This choice balances clustering quality and interpretability by providing finer subgroup differentiation, while still maintaining moderate *SW/SB* and *R*-squared values and relatively low DBI. Abbreviations: *SW*, standard deviation of the group; *SB*, standard deviation between groups

Table S6 Characteristics of lifestyle subgroups.

	***Mean* (*SD*)**					***Statistic***	
	Subgroup1 (*n* = 130)	Subgroup2 (*n* = 99)	Subgroup3 (*n* = 86)	Subgroup4 (*n* = 88)	Subgroup5 (*n* = 50)	χ^2/*F*-value	*p*-value
Demographic variables							
Age [a]	67.7 (7.0)	67.1 (6.5)	67.7 (6.8)	68.0 (6.4)	68.6 (5.8)	0.513	.727
Sex [b]	female, *n*= 49 (37.7%)	female, *n*= 48 (48.5%)	female, *n*= 48 (55.8%)	female, *n*= 41 (46.6%)	female, *n*= 27 (54.0%)	8.280	.082
Education [a]	5.88 (1.70)	6.74 (1.99)	6.49 (2.12)	6.59 (2.03)	5.86 (1.71)	4.235	**.002**
Lifestyle variables							
Packyears [c]	21.73 (20.88)	7.49 (14.89)	7.73 (15.24)	10.57 (23.36)	20.9 (24.15)	**23.265**	**<.001**
Alcohol [c]	13.84 (24.94)	11.26 (15.58)	4.27 (8.83)	12.75 (15.78)	7.73 (13.28)	**9.460**	**<.001**
Physical activity [c]	20.28 (29.21)	75.29 (64.51)	27.78 (19.02)	40.31 (33.12)	91.12 (85.60)	**56.53**	**<.001**
Stairs [c]	4.71 (8.26)	10.44 (9.01)	4.83 (5.22)	6.10 (8.48)	2.80 (4.62)	**16.576**	**<.001**
Social integration [c]	11.00 (5.02)	10.72 (4.83)	11.72 (3.23)	19.17 (7.16)	12.44 (5.27)	**39.008**	**<.001**
Visit_count [c]	4.76 (3.80)	4.39 (2.96)	5.1 (2.23)	10.94 (5.13)	6.16 (2.36)	**48.021**	**<.001**
Sporty_count [c]	1.50 (1.01)	3.66 (1.29)	2.47 (1.11)	2.60 (1.28)	3.00 (1.12)	**51.659**	**<.001**
Physical_count [c]	0.04 (0.19)	0.13 (0.44)	0.15 (0.42)	0.11 (0.49)	0.24 (0.59)	2.129	0.076
Cognitive variables							
Cognitive status [c]	14.35 (2.24)	15.05 (2.23)	15.07 (2.26)	14.97 (2.51)	14.36 (2.80)	0.847	.496
Language comprehension [c]	30.09 (5.22)	32.36 (4.29)	30.91 (5.24)	30.77 (5.32)	29.28 (5.31)	1.890	.111
Nomination [c]	14.49 (0.77)	14.67 (0.61)	14.45 (0.89)	14.50 (0.83)	14.36 (0.96)	0.957	.431
Figural memory [c]	7.55 (4.20)	9.77 (3.96)	7.69 (3.76)	8.08 (4.34)	7.60 (4.30)	**3.651**	**.006**

Verbal WM [c]	4.59 (1.10)	4.88 (1.18)	4.48 (0.99)	4.75 (1.15)	4.34 (0.80)	2.11	.079
Phonemic fluency [c]	18.49 (6.13)	19.78 (6.17)	19.09 (6.01)	19.12 (6.04)	18.40 (6.40)	0.194	.942
Phonemic switch [c]	17.83 (5.81)	20.07 (5.30)	19.64 (6.30)	18.15 (5.88)	17.38 (5.81)	**2.639**	**.033**
Semantic fluency [c]	22.43 (5.79)	24.88 (6.63)	23.34 (6.67)	23.49 (7.13)	21.54 (7.11)	1.902	.109
Semantic switch [c]	19.15 (4.13)	20.32 (4.77)	19.58 (4.68)	19.25 (4.82)	18.8 (5.58)	0.864	.486
Visuospatial WM [c]	4.66 (1.18)	4.72 (0.95)	4.53 (0.98)	4.65 (1.08)	4.36 (1.01)	0.795	.529
Episodic memory [c]	56.84 (12.55)	61.98 (9.56)	61.47 (10.91)	61.14 (11.91)	58.51 (10.93)	**3.007**	**.018**
Long-term memory [c]	10.25 (2.66)	11.19 (2.43)	11.23 (2.35)	10.41 (2.77)	10.23 (2.82)	1.943	.102
Executive control [c]	2.48 (0.99)	2.27 (0.69)	2.45 (0.99)	2.59 (1.12)	2.57 (0.93)	1.224	.300
MRI variables (in BF-Life$_{multi}$ feature set)							
mean GMV [d]	1283.57 (133.17)	1294.24 (129.86)	1311.52 (125.42)	1304.49 (148.89)	1289.49 (155.14)	**3.928**	**.004**
mean CT [d]	2.46 (0.15)	2.53 (0.12)	2.51 (0.12)	2.47 (0.14)	2.51 (0.13)	**5.710**	**<.001**
mean SA [d]	512.23 (52.65)	508.18 (52.45)	516.13 (49.25)	525.11 (55.12)	491.51 (48.29)	**6.996**	**<.001**
mean net FC [c]	0.34 (0.05)	0.36 (0.05)	0.35 (0.05)	0.35 (0.05)	0.36 (0.05)	**2.632**	**.034**
mean node FC [c]	0.64 (0.04)	0.63 (0.04)	0.64 (0.04)	0.64 (0.04)	0.61 (0.05)	**6.592**	**<.001**
mean FA [c]	0.356 (0.055)	0.359 (0.061)	0.350 (0.057)	0.365 (0.058)	0.385 (0.056)	**3.844**	**.004**
mean MD [c]	/	/	/	/	/	/	/
mean RD [c]	7.285×10^{-4} (1.738×10^{-4})	7.171×10^{-4} (1.456×10^{-4})	7.644×10^{-4} (1.734×10^{-4})	7.092×10^{-4} (1.366×10^{-4})	6.749×10^{-4} (1.017×10^{-4})	**3.595**	**.007**
mean AD [c]	/	/	/	/	/	/	/
mean ODI [c]	0.152 (0.042)	0.174 (0.042)	0.175 (0.046)	0.174 (0.039)	0.162 (0.042)	**4.961**	**<.001**
mean ICVF [c]	0.279 (0.098)	0.319 (0.085)	0.322 (0.093)	0.325 (0.075)	0.295 (0.095)	**4.608**	**.001**

Note: The mean value and standard deviation are represented as mean (*SD*). Significant group differences are indicated in bold. For MRI variables, we only compared between-group differences in the mean values of BF-Life$_{\mathbf{multi}}$ features across modalities. There is no feature of MD or AD in the BF-Life$_{\mathbf{multi}}$ model. Abbreviations: Packyears, packyears of smoking; Alcohol, alcohol consumption; Physical activity, measured by metabolic equivalent of the task; Stairs, flights of stairs climbed; Visit_count, count of visits from children, relatives, and friends; Sporty/Physical_count, count of sporty/physical activities; WM, working memory

[a] Univariate Analysis of Variance

[b] Pearson Chi-Square

[c] Univariate Analysis of Variance (controlling age, sex and education)

[d] Univariate Analysis of Variance (controlling age, sex, education, and estimated total intracranial volume)

[e] Quade nonparametric ANCOVA (controlling age, sex and education)

Table S7 Performance of the BF-Life$_{multi}$ model in predicting figural, episodic, and long-term memory.

Models	Targets	Algorithms	Median R^2				Median *MAE*			
			DISCOVERY baseline	TEST$_{CROSS}$ baseline	TEST$_{LONG}$ baseline	TEST$_{LONG}$ follow-up	DISCOVERY baseline	TEST$_{CROSS}$ baseline	TEST$_{LONG}$ baseline	TEST$_{LONG}$ follow-up
BF-Life$_{multi}$ models	Figural memory	ENR	0.13	0.12	-0.20	-0.15	3.16	3.35	3.30	3.32
		Ridge	0.13	0.11	-0.20	-0.15	3.18	3.36	3.30	3.33
		SVRrbf	0.09	0.08	-0.24	-0.19	3.21	3.45	3.36	3.46
		Lasso	0.09	0.09	-0.19	-0.15	3.22	3.42	3.27	3.34
		RVRlin	0.07	0.09	-0.33	-0.26	3.22	3.41	3.50	3.44
	Episodic memory	ENR	0.04	0.07	-0.06	-0.62	8.77	8.93	9.83	10.72
		Ridge	0.06	0.08	-0.08	-0.64	8.67	8.92	9.92	10.79
		SVRrbf	0.05	0.05	-0.07	-0.68	8.65	9.10	9.80	10.97
		Lasso	0.03	0.07	-0.07	-0.63	8.79	8.89	9.88	10.70
		RVRlin	-2.46×10^{-3}	0.07	-0.11	-0.63	8.92	8.93	9.97	10.69
	Long-term memory	ENR	0.04	0.05	0.04	-0.29	2.00	1.92	2.15	2.21
		Ridge	0.05	0.06	0.04	-0.30	1.97	1.91	2.16	2.20
		SVRrbf	0.01	0.04	0.03	-0.24	2.03	1.88	2.13	2.11
		Lasso	0.01	0.02	0.01	-0.31	2.06	1.98	2.19	2.22
		RVRlin	0.02	0.03	0.02	-0.33	2.05	1.91	2.18	2.23

Note: BF-Life$_{multi}$ was built based on a $k = 5$ clustering solution. Abbreviations: R^2, coefficient of determination; *MAE*, mean absolute error; BF-Life$_{multi}$, brain features derived from group contrasts based on multiple lifestyle profiles; ENR, elastic net regression; Ridge, ridge regression; SVRrbf, support vector regression with radial basis function kernel; Lasso, least absolute shrinkage and selection operator regression; RVRlin, relevance vector regression with the linear kernel

Table S8 Top 30% features in the BF-Life$_{multi}$ model in predicting figural memory using ENR and SHAP analysis.

**Parameters**	**Labels**	**Network/ fiber type**	**Mean |SHAP| value**	**Harvard-Oxford Cortical Structural Atlas**	**AAL3**	**Brodmann**	**Julich Brain Atlas**
GMV	**SAN_FrOperIns_2 (R)**	SAN	0.24	Insular Cortex (R), 92.84%	Insula (R), 80.44%	BA48 (R), 90.53%	Area Id6 (Insula), 42.29%
GMV	**DMN_Temp_5 (L)**	DMN	0.14	Temporal Pole (L), 66.64%	Temporal_Pole_Sup (L), 54.13%	BA38 (L), 44.95%	Area STS2 (STS), 17.96%
GMV	**DMN_PFC_13 (L)**	DMN	0.09	Superior Frontal Gyrus (L), 28.91%	Frontal_Sup_Medial (L), 97.33%	BA10 (L), 32.46%	Area p32 (pACC), 35.13%
GMV	**FPN_Temp_2 (R)**	FPN	0.05	Middle Temporal Gyrus, temporooccipital part (R), 42.31%	Temporal_Mid (R), 63.16%	BA20 (R), 51.70%	Temporal-to-Parietal (GapMap), 88.10%
GMV	**VIN_10 (R)**	VIN	0.04	Lateral Occipital Cortex, inferior division (R), 90.46%	Frontal_Inf_Oper (R), 64.87%	BA19 (R), 80.40%	Area hOc4v (LingG), 35.80%
CT	**SAN_TempOccPar_6 (R)**	SAN	0.21	Supramarginal Gyrus, anterior division (R), 82.32%	SupraMarginal (R), 87.10%	BA2 (R), 52.37%	Area PF (IPL), 59.05%
CT	**SMN_1 (R)**	SMN	0.14	Planum Polare (R), 39.56%	Temporal_Pole_Sup (R), 35.11%	BA48 (R), 38.26%	Area Te 3 (STG), 29.40%
CT	**SMN_9 (L)**	SMN	0.09	Precentral Gyrus (L), 54.16%	Rolandic_Oper (L), 60.00%	BA48 (L), 70.45%	Area Op4 (POperc), 28.55%
CT	DAN_PrCv_1 (R)	DAN	0.07	Precentral Gyrus (R), 59.60%	Frontal_Inf_Oper (R), 64.87%	BA44 (R), 47.06%	Area 6v3 (PreCG), 20.39%
CT	VIN_14 (L)	VIN	0.06	Lateral Occipital Cortex, inferior division (L), 86.17%	Occipital_Mid (L), 51.34%	BA19 (L), 42.99%	Area hOc4lp (LOC), 41.21%
CT	DMN_Temp_1 (L)	DMN	0.05	Middle Temporal Gyrus, anterior division (L), 38.12%	Temporal_Mid (L), 51.35%	BA20 (L), 52.39%	Temporal-to-Parietal (GapMap), 55.18%
CT	SAN_TempOccPar_5 (R)	SAN	0.04	Supramarginal Gyrus, anterior division (R), 36.67%	SupraMarginal (R), 69.97%	BA48 (R), 66.94%	Frontal-to-Occipital (GapMap), 26.5035%<br>CA1 (Hippocampus), 22.85% (551)

SA	**VIN_10 (R)**	VIN	0.15	Lateral Occipital Cortex, inferior division (R), 90.46%	Frontal_Inf_Oper (R), 64.87%	BA19 (R), 80.40%	Area hOc4v (LingG), 35.80%
SA	SMN_34 (L)	SMN	0.04	Superior Frontal Gyrus (L), 42.70%	Precentral (L), 32.57%	BA6 (L), 91.18%	Area 6d1 (PreCG), 60.24%
node FC	**VIN_20 (L)**	VIN	0.12	Cingulate Gyrus, posterior division (L), 40.90%	Calcarine (L), 51.42%	BA17 (L), 31.23%	Frontal-to-Occipital (GapMap), 78.23%
node FC	**SMN_8 (R)**	SMN	0.08	Superior Temporal Gyrus, posterior division (R), 59.20%	Temporal_Sup (R), 95.29%	BA22 (R), 63.16%	Temporal-to-Parietal (GapMap), 32.92%
node FC	**LIN_OFC_3 (R)**	LIN	0.07	Frontal Pole (R), 48.26%	Rectus (R), 54.86%		Area Fo1 (OFC), 53.58%
node FC	VIN_5 (R)	VIN	0.07	Lingual Gyrus (R), 62.57%	Lingual (R), 46.60%	BA37 (R), 65.61%	Area Ph1 (PhG), 44.02%
node FC	**DMN_PFC_6 (L)**	DMN	0.06	Frontal Pole (L), 99.25%	Frontal_Sup_2 (L), 78.57%	BA11 (L), 60.22%	Area Fp1 (FPole), 59.35%
node FC	VIN_3 (R)	VIN	0.05	Temporal Occipital Fusiform Cortex (R), 92.31%	Fusiform (R), 96.44%	BA37 (R), 89.85%	Area FG4 (FusG), 34.18%
node FC	SMN_7 (R)	SMN	0.04	Planum Temporale (R), 63.56%	Temporal_Sup (R), 91.40%	BA22 (R), 54.24%	Area TPJ (STG, SMG), 31.58%
net FC	**DMN_w**	DMN	0.05	/	/	/	/
FA	Posterior_corona_radiata (R)	Projection	0.10	/	/	/	/
RD	Cingulum_cingulate (R)	Association	0.06	/	/	/	/
ICVF	Superior_corona_radiata (L)	Projection	0.08	/	/	/	/
ICVF	Anterior_corona_radiata (L)	Projection	0.06	/	/	/	/
ICVF	Genu_of_corpus_callosum	Commissural	0.05	/	/	/	/
ICVF	External_capsule (L)	Association	0.05	/	/	/	/
ODI	Superior_longitudinal_fasciculus (R)	Association	0.08	/	/	/	/

Note: The row with blue color represents the common feature across all five algorithms. We determined the location of a region in the four atlases and the percentage of overlap. Abbreviations: L, left hemisphere; R, right hemisphere

Table S9 Sensitivity analysis: Performance of the BF-Life$_{multi}$ model using ENR under alternative clustering solutions.

Targets	Models	Median R^2				Median *MAE*			
		DISCOVERY baseline	TEST$_{CROSS}$ baseline	TEST$_{LONG}$ baseline	TEST$_{LONG}$ follow-up	DISCOVERY baseline	TEST$_{CROSS}$ baseline	TEST$_{LONG}$ baseline	TEST$_{LONG}$ follow-up
	**BF-Life$_{multi}$ models**								
Figural memory	$k = 2$	0.06	0.09	-0.15	-0.10	3.23	3.39	3.27	3.29
	$k = 3$	0.08	0.04	-0.24	-0.17	3.22	3.49	3.33	3.42
	$k = 4$	0.08	0.02	-0.29	-0.25	3.29	3.49	3.41	3.51
Episodic memory	$k = 2$	0.05	0.09	-0.11	-0.66	8.88	8.79	10.12	10.94
	$k = 3$	0.04	0.05	-0.07	-0.71	8.93	9.12	9.95	11.11
	$k = 4$	0.06	0.07	-0.07	-0.67	8.79	9.01	9.89	10.87
Long-term memory	$k = 2$	0.04	0.04	0.03	-0.31	2.03	1.96	2.16	2.21
	$k = 3$	0.02	0.04	0.07	-0.28	2.04	1.97	2.12	2.19
	$k = 4$	0.04	0.06	0.06	-0.29	2.04	1.95	2.10	2.18

Abbreviations: R^2, coefficient of determination; *MAE*, mean absolute error; ENR, elastic net regression; BF-Life$_{multi}$, brain features derived from group contrasts based on multiple lifestyle profiles; *k*, cluster number

Table S10 Sensitivity analysis: Performance of lifestyle-only reference models in predicting figural memory.

Algorithms	Median R^2		Median *MAE*	
	**DISCOVERY baseline**	**$TEST_{LONG}$ baseline**	**DISCOVERY baseline**	**$TEST_{LONG}$ baseline**
ENR	-5.51×10^{-4}	-0.19	3.44	3.33
Ridge	1.48×10^{-2}	-0.19	3.44	3.32
SVRrbf	-2.13×10^{-2}	-0.25	3.51	3.34
Lasso	3.24×10^{-3}	-0.19	3.45	3.34
RVRlin	1.80×10^{-2}	-0.19	3.42	3.32

Note: Models were trained using the original eight continuous lifestyle variables and evaluated in DISCOVERY and $TEST_{LONG}$ at baseline. $TEST_{CROSS}$ was not included because complete lifestyle-variable information was not available. Abbreviations: R^2, coefficient of determination; *MAE*, mean absolute error; ENR, elastic net regression; Ridge, ridge regression; SVRrbf, support vector regression with radial basis function kernel; Lasso, least absolute shrinkage and selection operator regression; RVRlin, relevance vector regression with the linear kernel

Table S11 Sensitivity analysis: Performance of the 11 models using ENR on the global cognitive component.

Models	Median R^2			Median *MAE*		
	**DISCOVERY baseline**	**$TEST_{CROSS}$ baseline**	**$TEST_{LONG}$ baseline**	**DISCOVERY baseline**	**$TEST_{CROSS}$ baseline**	**$TEST_{LONG}$ baseline**
**BF-$Life_{multi}$ models**						
k = 5	0.13	0.17	-0.05	1.66	1.67	1.61
BF-$Life_{single}$ **models**						
SMO	0.09	0.09	-0.02	1.68	1.76	1.60
ALC	0.12	0.12	-0.01	1.65	1.73	1.58
PA	0.08	0.05	-0.04	1.68	1.83	1.61
SI	0.11	0.11	0.03	1.66	1.75	1.55
**Data-driven model**						
BF_{data}-Var	0.13	0.17	0.05	1.61	1.71	1.50
BF_{data}-Match	0.17	0.17	0.08	1.58	1.70	1.48
BF_{data}-Var(Train)	0.17	0.19	0.02	1.60	1.67	1.53
BF_{data}-Match(Train)	0.16	0.19	0.02	1.61	1.67	1.53
**Incorporating non-brain data**						
BF-$Life_{multi}$ +dem: *k*=5	0.32	0.36	0.14	1.40	1.45	1.50
BF-$Life_{multi}$ +dem+life: *k*=5	0.36	/	0.19	1.37	/	1.47

Abbreviations: R^2, coefficient of determination; *MAE*, mean absolute error; ENR, elastic net regression; BF-$Life_{multi}$, brain features derived from group contrasts based on multiple lifestyle profiles; *k*, cluster number; BF-$Life_{single}$, brain features derived from group contrasts based on a single lifestyle factor; SMO / ALC / PA / SI: BF-$Life_{single}$ models based on smoking, alcohol consumption, physical activity, and social integration, respectively; BF_{data}, data-driven PCA brain features; Var = 80% variance explained; Match = matched feature number; [Train] = PCA applied in training set exclusively; +dem, includes demographic data; +dem+life, includes demographic and lifestyle data

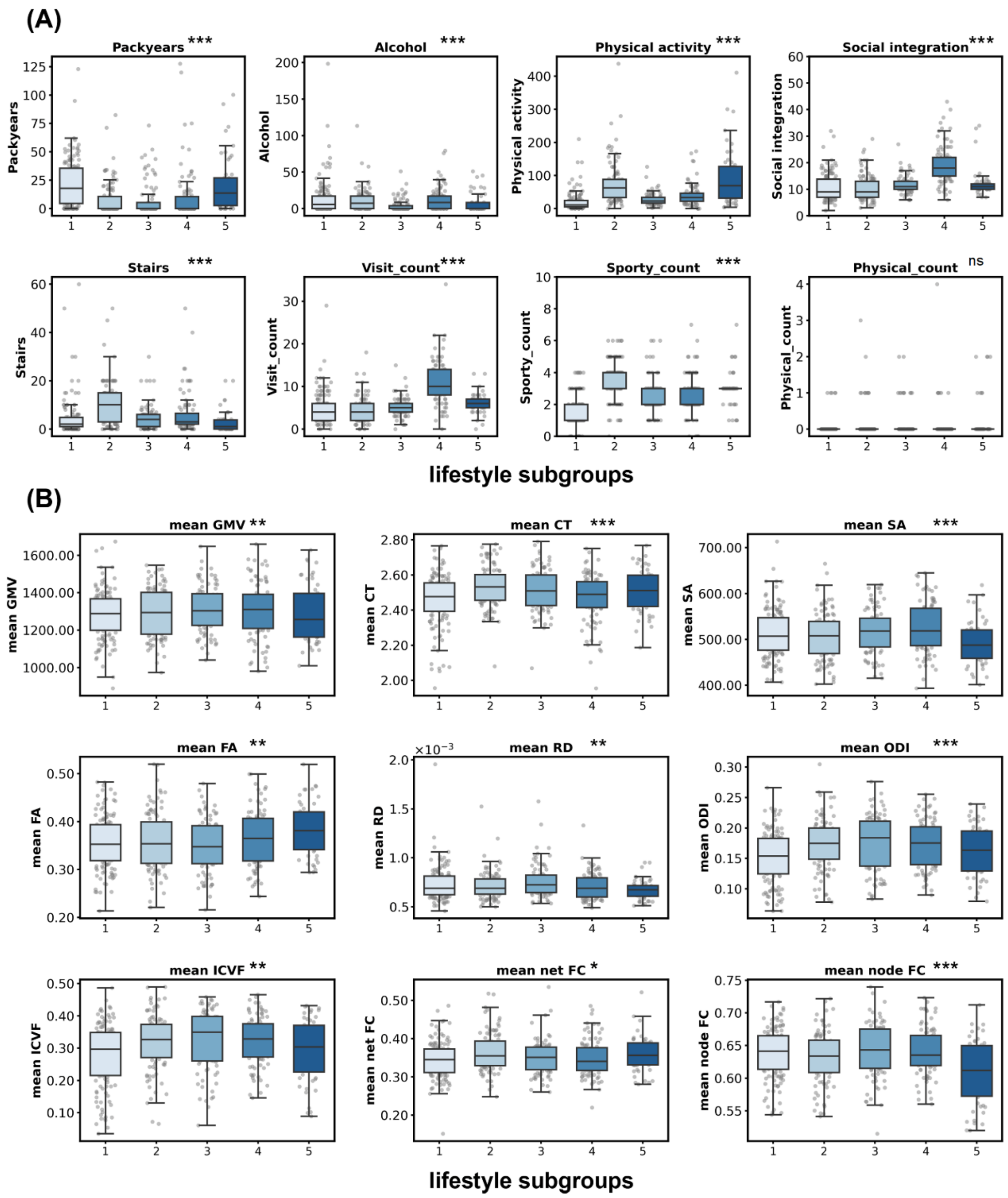

Figure S1 Lifestyle (A) and brain (B) profiles of each lifestyle subgroup. The asterisk (*) indicates a significant difference between groups. *, $p < .05$, **, $p < .01$, ***, $p < .001$. Abbreviations: Physical activity, measured by metabolic equivalent of the task; Stairs, flights of stairs climbed; Visit_count, count of visits from children, relatives, and friends; Sporty_count, count of sporty activities; Physical_count, count of physical activities

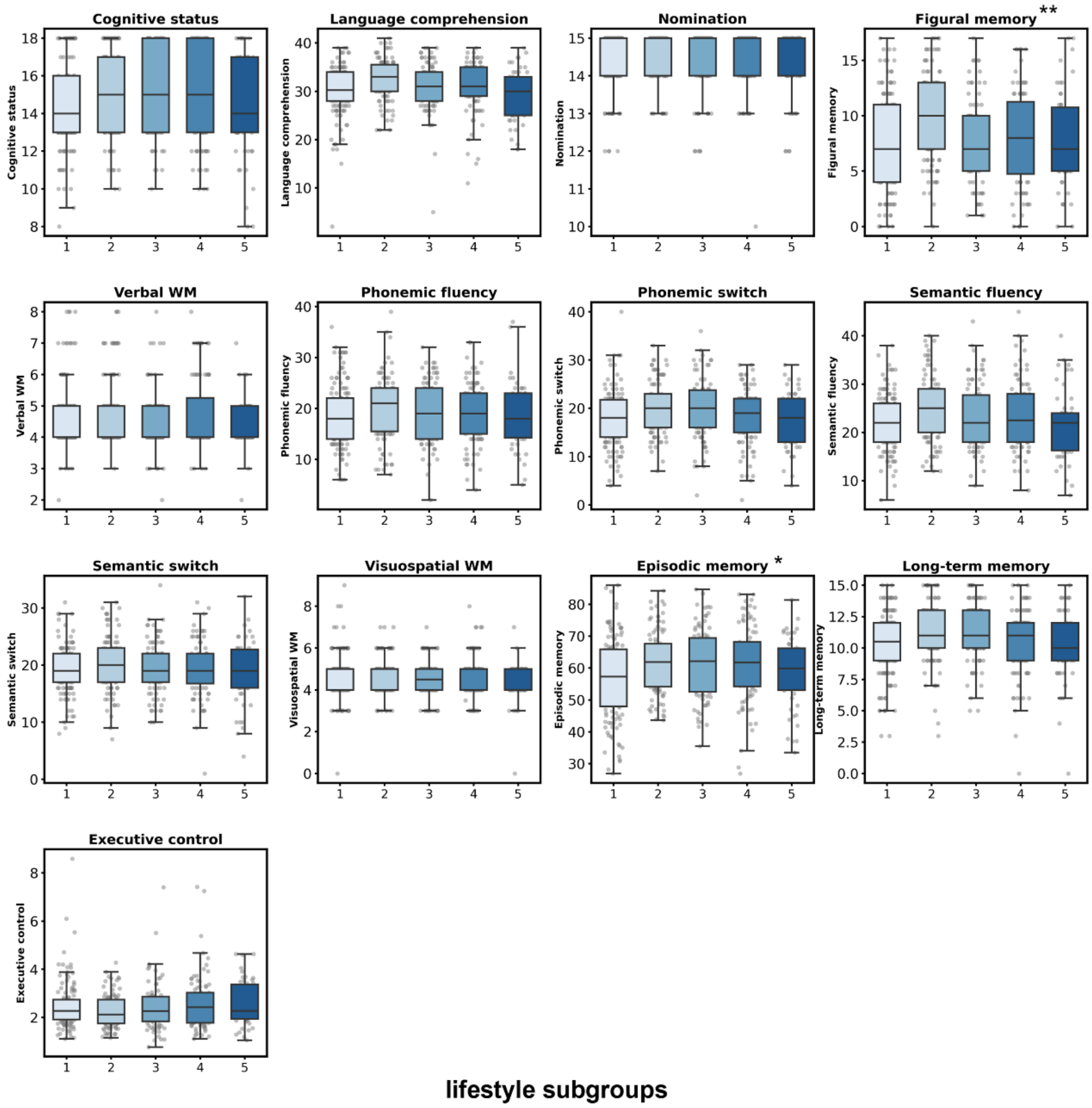

Figure S2 Cognitive profiles of each lifestyle subgroup. The asterisk (*) indicates a significant difference between groups. *, $p < .05$, **, $p < .01$. Abbreviations: WM, working memory

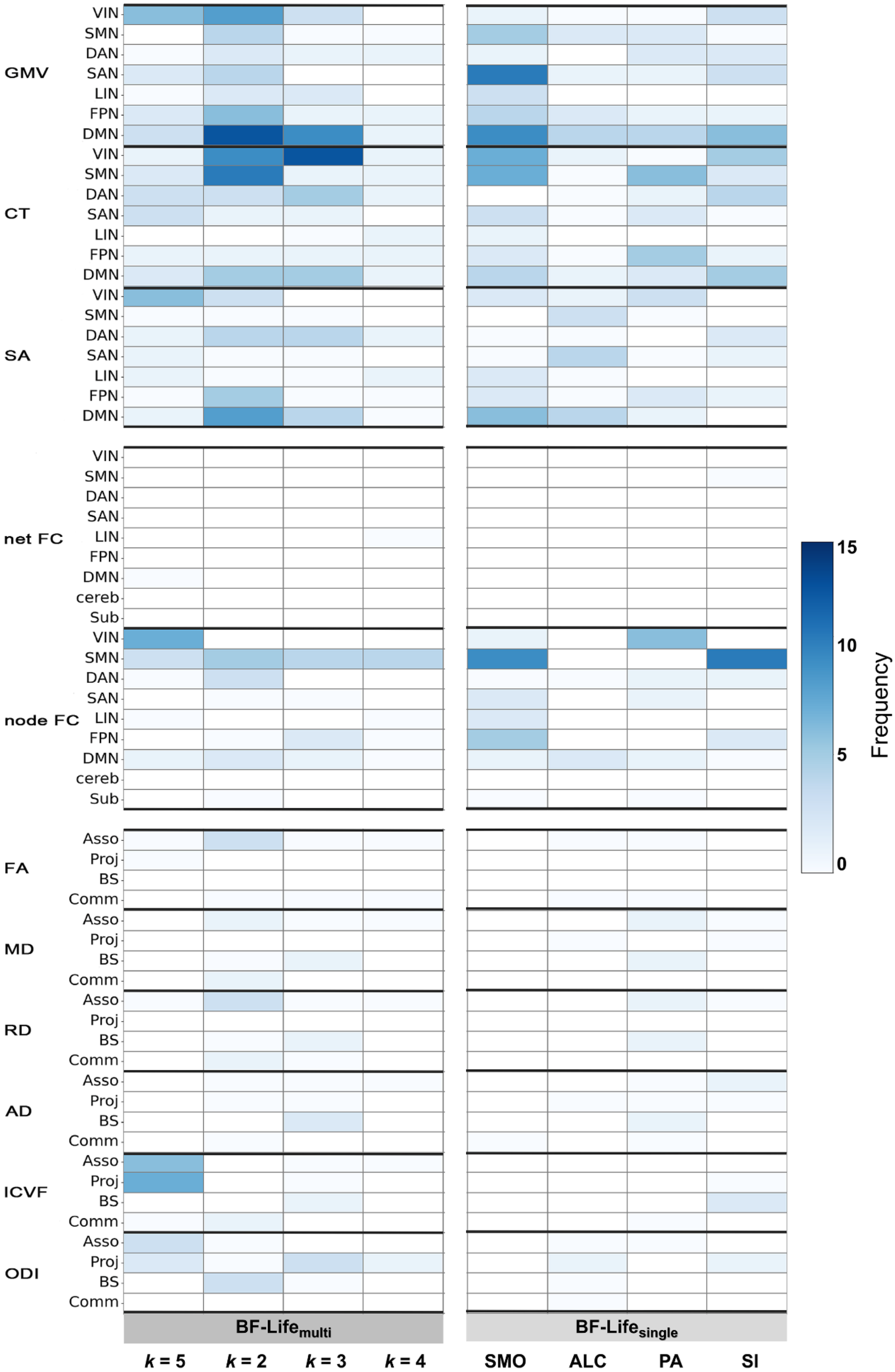

Figure S3 Feature selection frequency in the BF-Life$_{multi}$ and BF-Life$_{single}$ models, grouped by feature category. Abbreviations: k, cluster number; BF-Life$_{multi}$, brain features derived from group contrasts based on multiple lifestyle profiles; BF-Life$_{single}$, brain features derived from group contrasts based on a single lifestyle factor; SMO / ALC /

PA / SI: BF-Life$_{single}$ models based on smoking, alcohol consumption, physical activity, and social integration, respectively; GMV, gray matter volume; CT, cortical thickness; SA, surface area; FC, functional connectivity; net FC, network-wise FC related metrics; node FC, node-wise functional segregation; FA, fractional anisotropy; MD, mean diffusivity; AD, axial diffusivity; RD, radial diffusivity; ICVF, intracellular volume fraction; ODI, orientation dispersion index; Asso /Proj /BS /Comm, association /projection /brainstem /commissural fiber tract

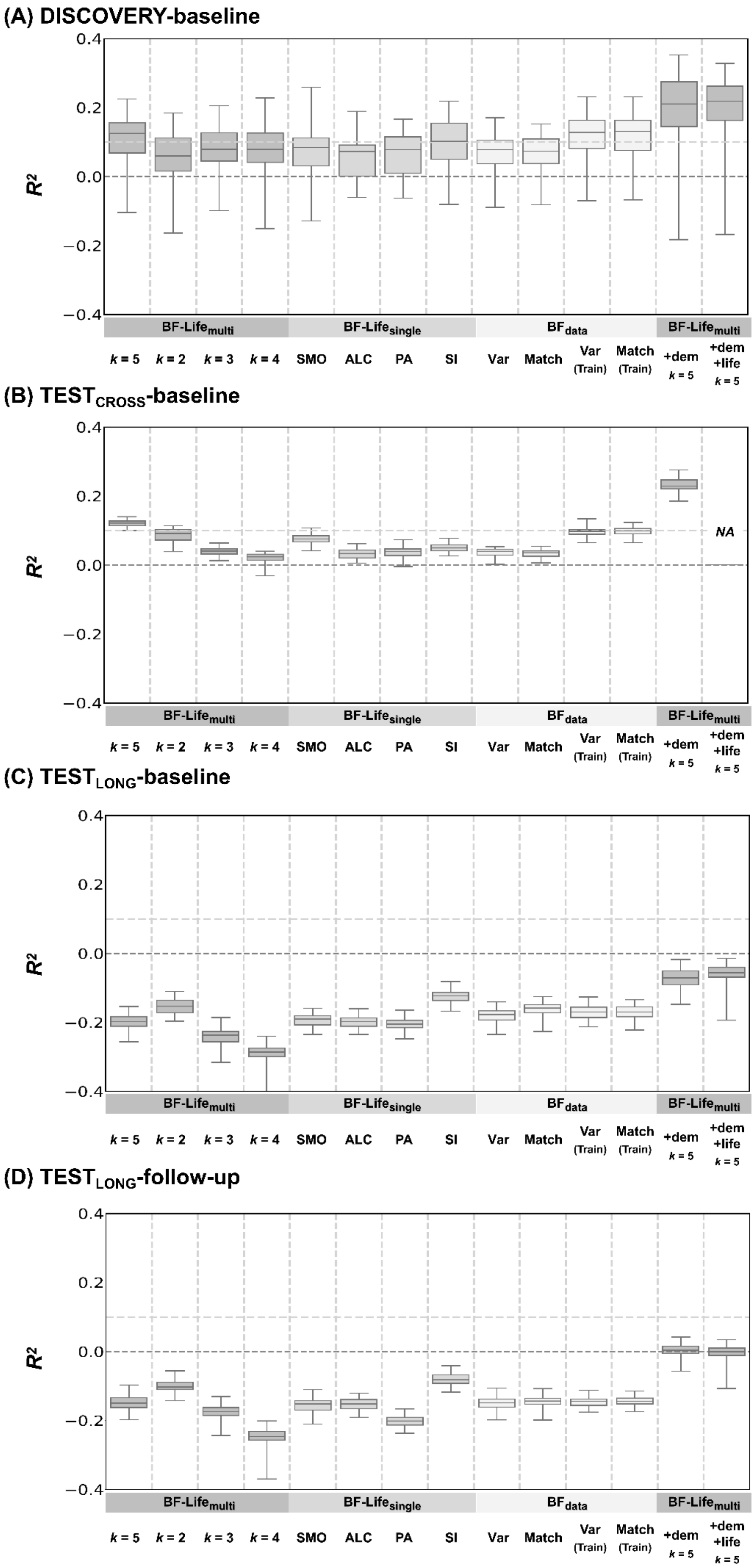
(A) DISCOVERY-baseline
(B) TEST$_{CROSS}$-baseline
(C) TEST$_{LONG}$-baseline
(D) TEST$_{LONG}$-follow-up
R^2
0.4
0.2
0.0
−0.2
−0.4
NA
BF-Life$_{multi}$
BF-Life$_{single}$
BF$_{data}$
BF-Life$_{multi}$
k = 5
k = 2
k = 3
k = 4
SMO
ALC
PA
SI
Var
Match
Var (Train)
Match (Train)
+dem k = 5
+dem +life k = 5

Figure S4 Predictive performance of various models for figural memory using ENR. The BF-Life$_{\mathbf{multi}}$ +dem+life model was not applied to the TEST$_{\text{CROSS}}$ dataset (**panel B**) due to missing lifestyle data and is marked as not available (NA). The light gray dashed line indicates an R^2 value of 0.10. Abbreviations: R^2: Coefficient of determination; *k*: Cluster number; BF-Life$_{\mathbf{multi}}$, brain features derived from group contrasts based on multiple lifestyle profiles; BF-Life$_{\mathbf{single}}$, brain features derived from group contrasts based on a single lifestyle factor; SMO / ALC / PA / SI: BF-Life$_{\mathbf{single}}$ models based on smoking, alcohol consumption, physical activity, and social integration, respectively; BF$_{\text{data}}$, data-driven PCA brain features; Var = 80% variance explained; Match = matched feature number; [Train] = PCA applied in training set exclusively; +dem, includes demographic data; +dem+life, includes demographic and lifestyle data

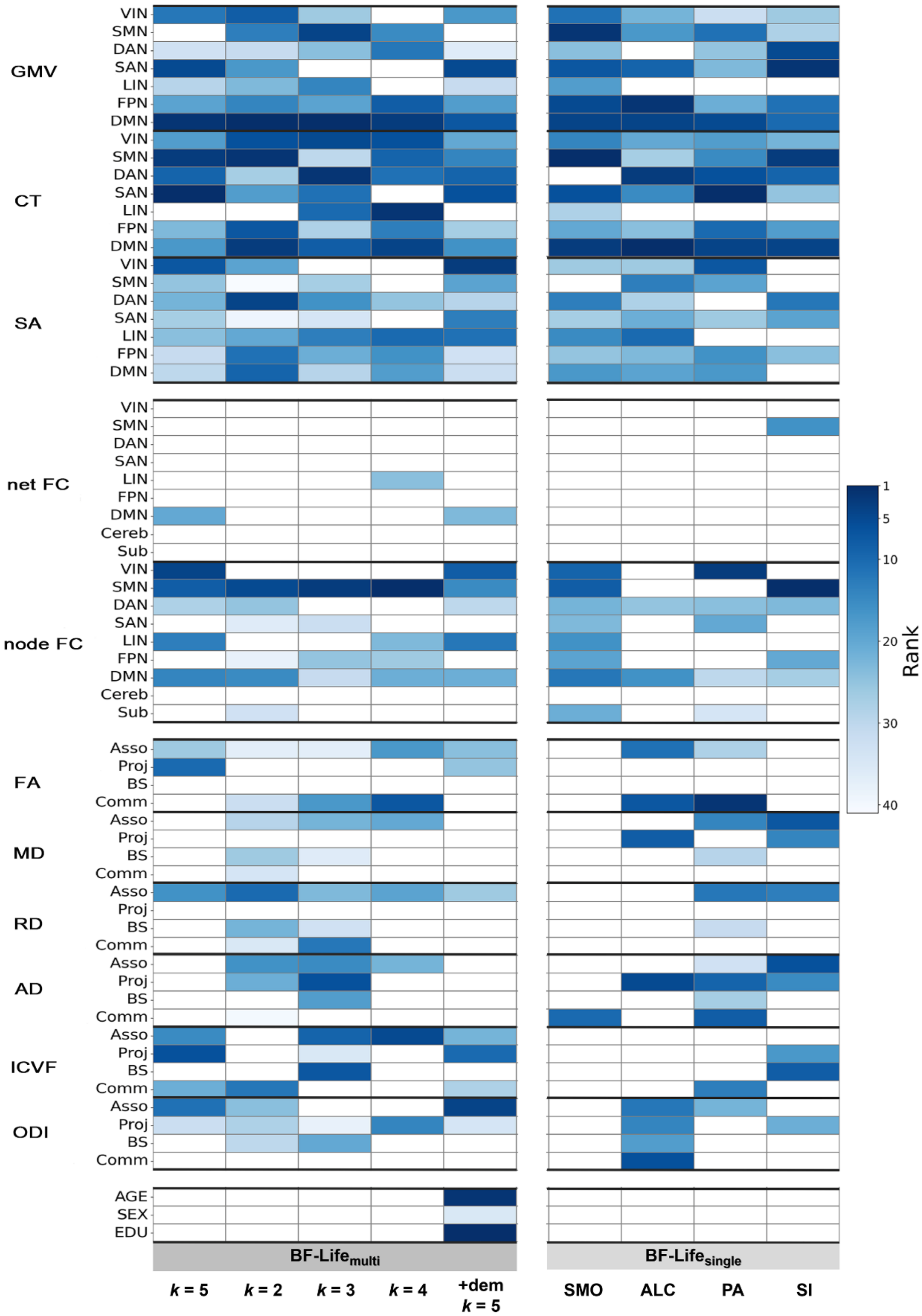

Figure S5 Importance of feature categories in nine models predicting figural memory using ENR in the TEST$_{CROSS}$ dataset. The heatmap shows values representing the sum of average absolute SHAP values over 50 folds for each category, arranged in descending order (dark blue = high, light blue = low). White cells indicate no features available in that model. Abbreviations: ENR, elastic net regression; BF-Life$_{multi}$, brain features derived from group contrasts based on multiple lifestyle profiles; BF-Life$_{single}$, brain features derived from group contrasts based on a single lifestyle factor; SMO / ALC / PA / SI: BF-Life$_{single}$ models based on smoking, alcohol consumption,

physical activity, and social integration, respectively; +dem, includes demographic data; GMV, gray matter volume; CT, cortical thickness; SA, surface area; FC, functional connectivity; net FC, network-wise FC-related metrics; node FC, node-wise functional segregation; FA, fractional anisotropy; MD, mean diffusivity; AD, axial diffusivity; RD, radial diffusivity; ICVF, intracellular volume fraction; ODI, orientation dispersion index; Asso /Proj /BS /Comm, association /projection /brainstem /commissural fiber tract; EDU, education

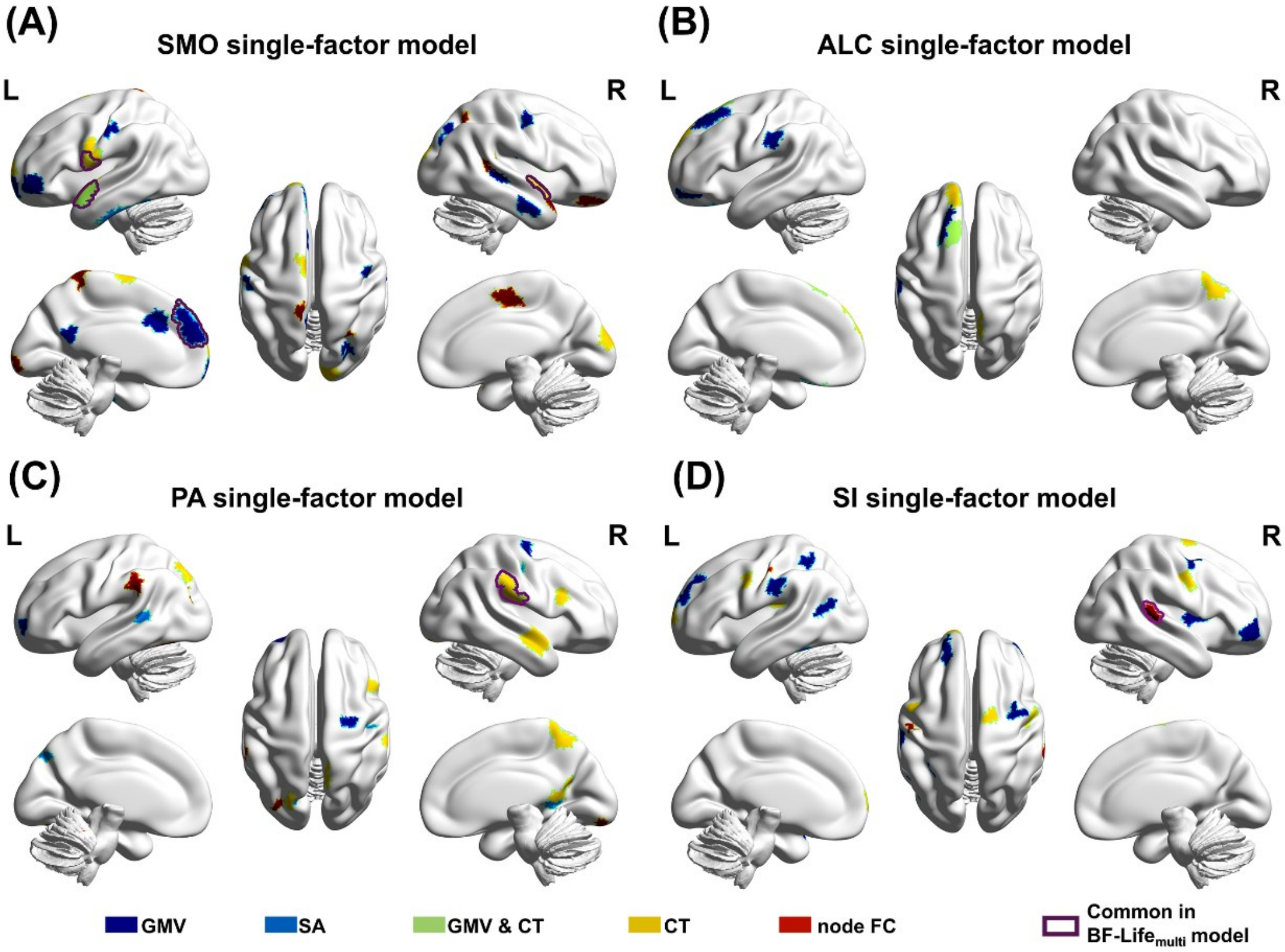

Figure S6 Feature importance in predicting figural memory using single lifestyle factor models and SHAP analysis with ENR. The brain regions marked by solid purple lines are important brain regions common to the BF-Life$_{\mathbf{multi}}$ model. Abbreviations: ENR, elastic net regression; BF-Life$_{\mathbf{single}}$, brain features derived from group contrasts based on a single lifestyle factor; SMO / ALC / PA / SI: BF-Life$_{\mathbf{single}}$ models based on smoking, alcohol consumption, physical activity, and social integration, respectively; GMV, gray matter volume; CT, cortical thickness; SA, surface area; FC, functional connectivity; FC segregation, node-wise functional segregation; L, left hemi sphere; R, right hemisphere; see Abbreviations List for full abbreviations

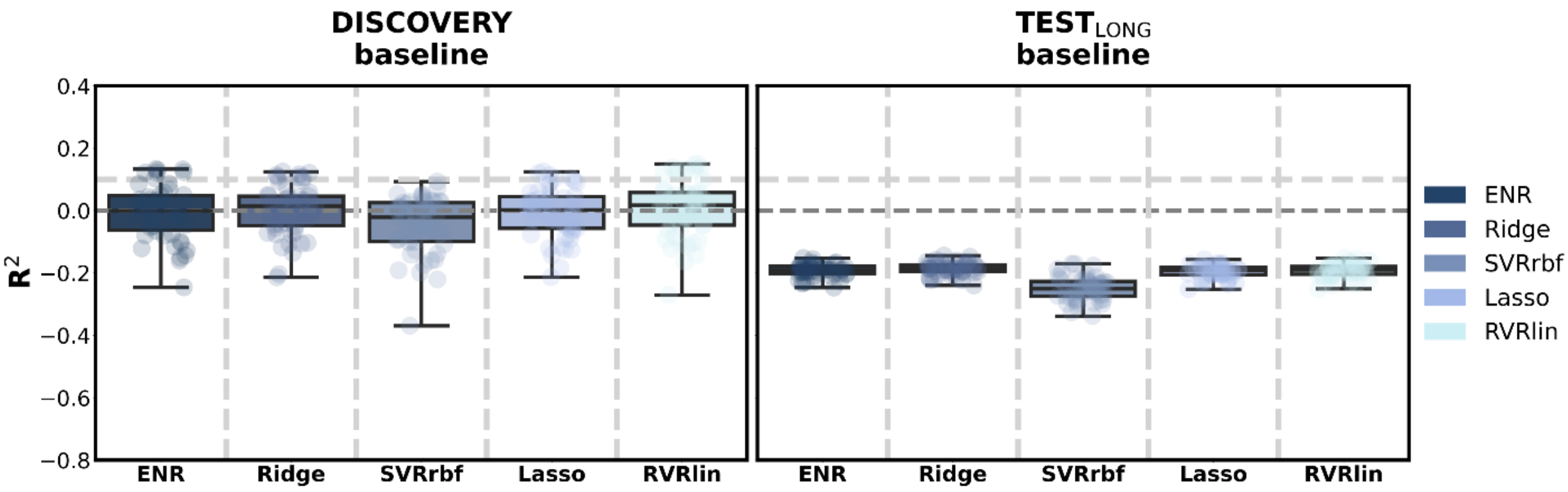

Figure S7 Predictive performance of lifestyle-only reference models using five machine learning algorithms. Models were trained using the original eight continuous lifestyle variables and evaluated in DISCOVERY and $TEST_{LONG}$ at baseline. $TEST_{CROSS}$ was not included because complete lifestyle-variable information was not available. The light gray dashed line indicates an R^2 value of 0.10. Abbreviations: ENR, elastic net regression; Ridge, ridge regression; SVRrbf, support vector regression with RBF kernel; Lasso, least absolute shrinkage and selection operator regression; RVRlin, relevance vector regression with linear kernel

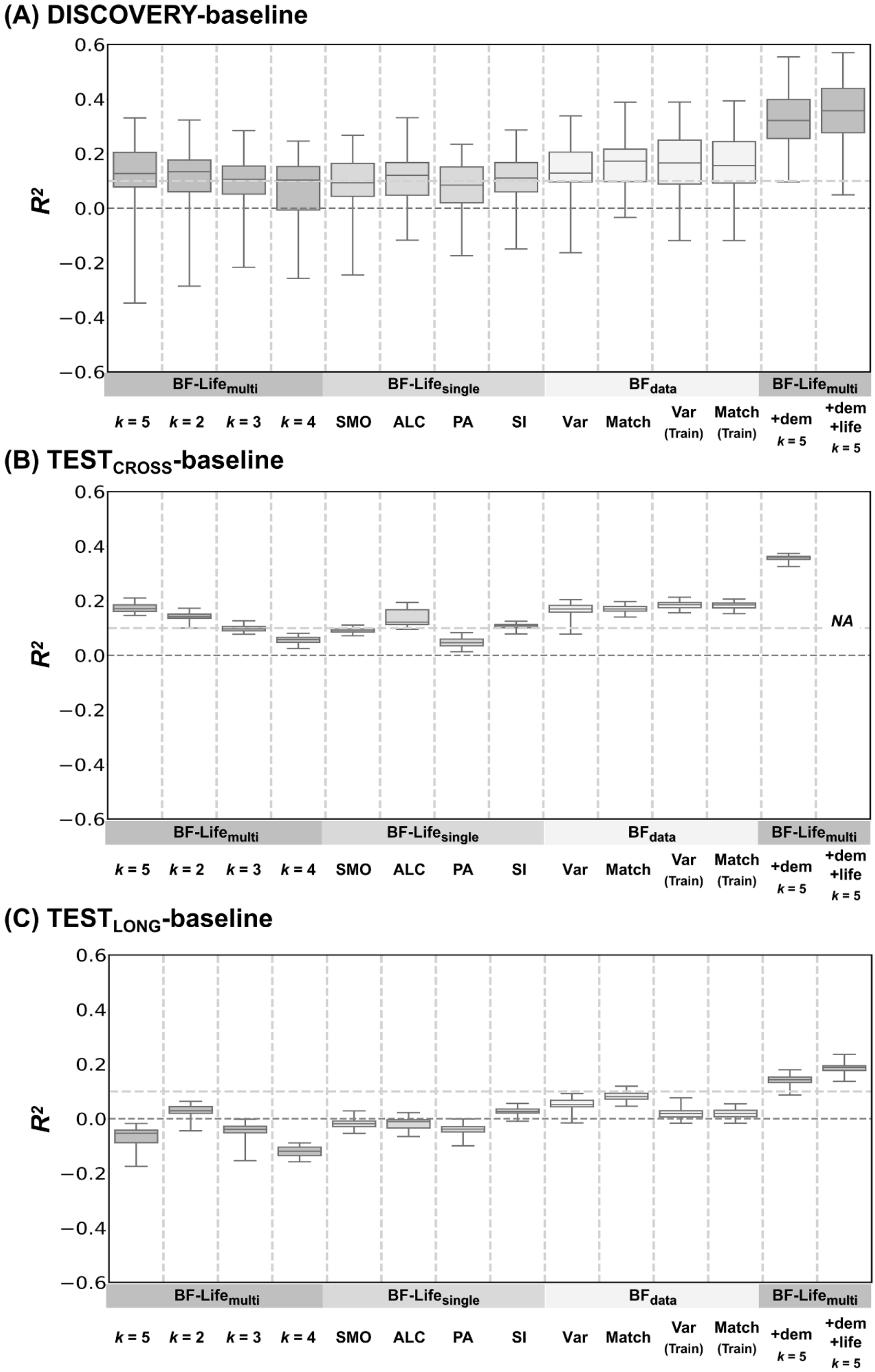

Figure S8 Predictive performance of various models for the global cognitive component using ENR. The BF-

$Life_{multi}$ +dem+life model was not applied to the $TEST_{CROSS}$ dataset (**panel B**) due to missing lifestyle data and is marked as not available (NA). The light gray dashed line indicates an R^2 value of 0.10. Abbreviations: ENR, elastic net regression; R^2: Coefficient of determination; *k*: Cluster number; BF-$Life_{multi}$, brain features derived from group contrasts based on multiple lifestyle profiles; BF-$Life_{single}$, brain features derived from group contrasts based on a single lifestyle factor; SMO / ALC / PA / SI: BF-$Life_{single}$ models based on smoking, alcohol consumption, physical activity, and social integration, respectively; BF_{data}, data-driven PCA brain features; Var = 80% variance explained; Match = matched feature number; [Train] = PCA applied in training set exclusively; +dem, includes demographic data; +dem+life, includes demographic and lifestyle data

eReferences

1 Kalbe, E. *et al.* DemTect: a new, sensitive cognitive screening test to support the diagnosis of mild cognitive impairment and early dementia. *Int J Geriatr Psych* **19**, 136–143 (2004). https://doi.org/10.1002/gps.1042

2 Schmidt, K. H. & Metzler, P. Wst-Wortschatztest. *Göttingen: Beltz Test* **16** (1992).

3 Morris, J. C. *et al.* The Consortium to Establish a Registry for Alzheimer's Disease (CERAD). Part I. Clinical and neuropsychological assessment of Alzheimer's disease. *Neurology* **39**, 1159–1165 (1989). https://doi.org/10.1212/wnl.39.9.1159

4 Benton, A. L., Benton-Sivan, A., Spreen, O. & Streck, P. Der Benton-Test. *Göttingen: Verlag Hans Huber.* (2009).

5 Oswald, W. D. & Fleischmann, U. M. Nürnberger-Alters-Inventar (NAI). *Göttingen: Hogrefe.* (1997).

6 Aschenbrenner, S., Tucha, O. & Lange, K. W. Der Regensburger Wortflüssigkeits-Test (RWT). *Göttingen: Hogrefe.* (2000).

7 Schelling, D. Block-Tapping-Test. *Frankfurt: Swets Test Services* (1997).

8 Lux, S., Hartje, W., Reich, C. & Nagel, C. VGT: Verbaler Gedächtnistest: Bielefelder Kategorielle Wortlisten. *Göttingen: Verlag Hans Huber* (2012).

9 Caspers, S. *et al.* Studying variability in human brain aging in a population-based German cohort-rationale and design of 1000BRAINS. *Front Aging Neurosci* **6**, 149 (2014). https://doi.org/10.3389/fnagi.2014.00149
